# Do Young Children Use Verbal Disfluency as a Cue to Their Own Confidence?

**DOI:** 10.1111/desc.13617

**Published:** 2025-03-05

**Authors:** Eloise West, Carolyn Baer, Lisa Yu, Darko Odic

**Affiliations:** ^1^ Department of Psychology University of British Columbia Vancouver Canada; ^2^ Department of Psychology University of British Columbia & Algoma University Brampton Canada

**Keywords:** confidence, metacognition, processing fluency, verbal disfluency

## Abstract

Metacognitive reasoning is central to decision‐making. For every decision, we can also judge our trust in that decision, or our level of *confidence*. The mechanisms and representations underlying reasoning about confidence remain debated. We test whether children rely on *processing fluency* to infer their own confidence: do decisions that come quickly and easily lead to high confidence, while decisions that are slow and effortful result in low confidence? Using children's verbal disfluency—fillers (e.g., “umm,” “uhh”), hedges (e.g., “I think,” “maybe”), and pauses in speech—as an observable index of processing fluency, we assess whether children's reports of confidence are a read‐out of their verbal disfluency. Five‐to‐eight‐year‐olds answered semantic questions about animals and performed perceptual comparisons, then reported their confidence in their answers in a two‐alternative forced‐choice confidence judgment task. Verbal disfluency predicted both answer accuracy and children's reports of confidence: children produced more fillers, more hedges, and longer speech onsets during incorrect trials and during low confidence trials. But we also found a dissociation between fluency and confidence. When examining trials where accuracy and confidence diverge (i.e., correct but low confidence or incorrect but high confidence trials), we observe no reliable relationship between confidence and fillers and hedges, and children take *longer* to begin answering on high confidence trials. We conclude that—in 5–8‐year‐old‐children—fluency is a reliable tracker of *accuracy* but not confidence, and that fluency is only predictive of metacognitive judgments in children when confidence and accuracy are aligned.

## 1 Introduction

Representations and decisions are marked by uncertainty. Everyday judgments like “how many apples are in this bowl?” or “did I remember to feed the cat?” are accompanied by a subjective *feeling* of evidence strength: a sense of belief or trust in our cognition and decisions (Grimaldi, Lau, and Basso 2015). It is no surprise, then, that modern theories of decision‐making and learning frequently incorporate estimates of uncertainty into their models (e.g., see Baer and Kidd 2022; Ma and Jazayeri 2014 for reviews), and that developmental theories in topics as broad as theory of mind, reading, and problem solving all require introspection about uncertainty for children to reason in adult‐like ways (Kuhn 2000).

**Summary**

- Five‐to‐eight‐year‐olds answered questions about animals and performed numerical comparisons, then rated their confidence in their answers using a two‐alternative forced‐choice.
- Children produced more verbal disfluency (fillers, e.g., “umm,” hedges, e.g., “maybe,” and longer pauses) during answers they judged to be low confidence.
- Despite the correlation between disfluency and confidence, we observed no reliable relationship when separating trials where accuracy and confidence dissociate.
- Children's level of confidence is not entirely determined from the amount of verbal disfluency they produced while answering.

How do we recognize if a thought, memory, or decision is reliable and trustworthy? The mechanisms and representations underlying judgments of confidence remain hotly debated (e.g., see Rahnev 2021 for a review). One popular idea is that confidence relies on observing the processing fluency of our own thoughts: when ideas, memories, and judgments come quickly and easily, we are more likely to believe them to be true and reliable (e.g., Alter and Oppenheimer 2009; Koriat 1993). This theory is supported by findings that reaction time or experimental manipulations of fluency (e.g., question difficulty, perceptual noise, and priming) are all reliable correlates of confidence judgments (Ackerman and Koriat 2011; Alter and Oppenheimer 2009).

Evidence for a link between fluency and confidence also comes from “verbal disfluency”—fillers (e.g., “umm,” “uhh”), hedge phrases (e.g., “I think,” “maybe”), and delays and pauses in speech. For example, Smith and Clark (1993) asked participants factual questions in a conversational setting (e.g., “in which sport is the Stanley Cup awarded?”), and after answering each question, participants used a Likert scale to indicate the likelihood they would be able to recognize the correct answer. Participants’ confidence judgments tracked their verbal disfluency, they produced more fillers, more hedges, and took longer to begin answering on trials where they indicated lower confidence. The broader theories on fluency and confidence argue that this relationship is causal: participants introspectively observe the fluency with which they generate responses and, when it is low, are subjectively less confident in their own answers (e.g., Alter and Oppenheimer 2009; Koriat 1993).

Children as young as two also show some understanding of the association between fluency and confidence: toddlers are less likely to learn from or imitate speakers who produce more verbal disfluency (Birch, Akmal, and Frampton 2010; Orena and White 2015), suggesting they perceive *others’* disfluency as a cue to unreliability (though see Kidd et al. 2011). Like adults, toddlers take longer to answer, and shift attention between response options on trials they ultimately answer incorrectly (Leckey et al. 2020). Krahmer and Swerts (2005) showed that Dutch‐speaking 7–8‐year‐olds produce more verbal disfluency on low confidence trials (see also Hübscher, Vincze, and Prieto 2019). Fluency might, therefore, be a critical and causal cue that both adults and young children use to determine their own confidence (Geurten, Willems, and Lloyd 2021; Guttentag and Dunn 2003).

Despite the wealth of data showing a predictive relationship between verbal fluency and confidence, an important mechanistic question remains. Fluency is itself correlated with a range of other cognitive processes that themselves may be the actual causes of confidence judgments. For example, imprecision in perceptual representations might lead to *both* high disfluency and low confidence, but from independent mechanisms—disfluency may occur while the observer is waiting for evidence to accumulate, but confidence may stem from a retrospective decision of whether the same choice would be made again (Pleskac and Busemeyer 2010). Indeed, some theories of metacognition have suggested that confidence is an accumulation of many separate cues—representational precision, one's beliefs about their ability on the task, estimates of how much attention was paid, and so forth (Pouget et al. 2016)—all of which may also increase disfluency, making it correlationally (but not causally) related to confidence.

Here, we directly examine the relationship between behavioral correlates of fluency and young children's (5‐8‐year‐olds) confidence. Fluency was indexed as verbal disfluency, as it can be easily recorded and quantified with minimal equipment. Verbal disfluency is also an ideal candidate measure of fluency as it occurs spontaneously and early in development (Ambrose and Yairi 1999; DeJoy and Gregory 1985), and is observed cross‐linguistically (Tian, Maruyama, and Ginzburg 2017). Following Smith and Clark (1993), we chose a broad set of verbal disfluency cues—fillers (e.g., “umm,” “uhh”), hedge phrases (e.g., “I think,” “maybe”), and delays and pauses in speech—testing them both cumulatively and separately.

We aim to isolate fluency from other decision‐making processes, and separate children's subjective confidence from the objective accuracy of their answers to evaluate whether fluency is a better metric of children's ability to answer, or their belief in their answers. To help identify trials where confidence and accuracy dissociate, we rely on a *relative confidence task*, in which children are given two questions successively, and then asked to indicate which of the two questions they are *more* confident on (Baer and Odic 2019; Mamassian 2020). The relative task does not require children to assign a number to their level of confidence in each individual answer, but instead has them compare *two* answers and decide which one they are more confident in. Previous work has shown this method to be an excellent metric for children's confidence decisions that controls for biased responding while adding minimal cognitive and memory load (Baer, Gill, and Odic 2018; Baer and Odic 2019, 2020). If verbal disfluencies are a cue for confidence in children, we should find that they reliably track their confidence choice: children should choose the trial on which they produced fewer verbal disfluencies as their “more confident” answer. This forced‐choice design allows us to especially triangulate on trials where accuracy and confidence dissociate (“discordant” trials: correct but rejected, and incorrect but chosen). If verbal disfluencies are a dominant and causal confidence cue, independent of their relation to answer accuracy, then we should find that they predict confidence even on trials where children are discordant. Alternatively, if verbal disfluencies are an index of accuracy but not metacognition, then they should predict confidence choice only on “concordant” trials where accuracy and confidence align.

The relative confidence method also alleviates the common bias toward overconfidence when using traditional verbal reports and Likert scale metacognitive judgment tasks (e.g., Bayard et al. 2021; Finn and Metcalfe 2014; van Loon et al. 2017). The overconfidence bias introduces complications in typical studies on fluency and confidence. For example, in a previous study of disfluency, Catalan‐speaking preschoolers indicated they felt “very sure” about a majority of answers (Hübscher, Vincze, and Prieto 2019). Researchers then observed a dissociation between disfluency (e.g., body, face, prosodic, and verbal cues) and confidence: although these children produced more disfluency during trials they answered incorrectly, they often explicitly reported high confidence despite producing disfluency. The problem here, however, is that the dissociation might stem from the tendency for children to just report high confidence on almost every trial, reducing the resolution of the data.

We selected 5‐to 8‐year‐olds for this study because children in this age range can reliably report confidence using the forced‐choice method (e.g., Baer and Odic 2019), and produce a variety of verbal disfluency. Hübscher, Vincze, and Prieto (2019) found developmental change in behavioral signaling of uncertainty: while children of all ages produced disfluency, 3‐year‐old children relied primarily on nonverbal (gestural and prosodic) cues and fillers (“umm,” “uhh”), and older children recruited a broader repertoire. By 5 years, children began using lexical markers of uncertainty, such as hedges (“I think,” “maybe”), though less frequently than adults, and it is important to note that their disfluencies largely did not track their reported confidence (Hübscher, Vincze, and Prieto 2019). Interestingly, 7–8‐year‐olds in Krahmer and Swerts (2005) produced longer speech onsets and rising intonation on low confidence trials, but they observed no association between filler disfluencies and confidence. Whether these older participants favor lexical markers of uncertainty remains unclear. We therefore further assess the development of cues to uncertainty, and differences between lexical and nonlexical disfluency. We separate categories of disfluency (fillers, hedges, and speech onsets) as independent predictors, and aim to disentangle these developmental trajectories and their relationship to confidence. Combined with a sensitive, age‐appropriate measure of confidence, this will allow us to determine whether the divergent findings among 3‐5‐year‐olds of Hübscher, Vincze, and Prieto (2019) and 7‐8‐year‐olds of Krahmer and Swerts (2005) reflect developmental differences in how fluency guides confidence, or instead methodological artifacts of measures of disfluency and metacognition.

A final consideration in our design is that fluency‐based accounts are more prominent in meta‐memory and meta‐reasoning than in meta‐perceptual literatures, and there is recent work that suggests that confidence sensitivity in these domains may differ in childhood (Baer, Ghetti, and Odic 2021). If true, it may be that fluency is a more relevant cue for some kinds of decisions (like memory and reasoning decisions) than others. We therefore also include both perceptually‐based decisions (“which side has more dots”; e.g., Halberda and Feigenson 2008) and semantically‐based decisions (“what animal is this” and “what sound does this animal make”), to investigate whether children use fluency as a cue differently across domains.

In sum, we adapt Smith and Clark's (1993) work in adults to be suited for English‐speaking children and examine: (1) how often 5–8‐year‐old English‐speaking children produce verbal disfluencies, and what types of disfluencies they produce; (2) whether their disfluencies reliably predict accuracy for both semantic‐ and perceptually‐based questions (e.g., “What colour is a hippo's milk” and “Which side has more dots?”); (3) whether their disfluencies predict confidence judgments; and, most importantly, (4) whether disfluencies predict metacognitive judgments even when confidence and accuracy dissociate. These questions are tested in a forced‐choice confidence design that eliminates overconfidence bias while adding minimal cognitive and memory load, and for both semantic and perceptual decisions, to examine if fluency acts differently as a marker of confidence across domains.

## 2 Methods

### 2.1 Open Data

All the coded data, stimuli, and programs used to collect the responses are available online at https://osf.io/6g2w4/?view_only=1d3179c016c2467395aef84f233d59aa. The raw audio recordings collected during the study cannot be posted due to privacy concerns, but the available coded data is a full transcription of each trial for each participant. The study's analyses were not preregistered.

### 2.2 Participants

Sixty children ages 5 to 8 years old participated in the study, with 15 children in each age group (5;0–5;11, *M* = 5.42 years, SD = 0.27, 7 boys, 8 girls; 6;0–6;11, *M* = 6.40 years, SD = 0.31, 7 boys, 7 girls, 1 nonbinary; 7;0–7;11, *M* = 7.43 years, SD = 0.23, 11 boys, 4 girls; 8;0–8;11, *M* = 8.51 years, SD = 0.24, 7 boys, 8 girls). Given this study involved understanding and answering questions in English, participants were required to hear at least 50% English in their daily life—validated by parent report via an online form. An additional 5 participants were tested but were excluded from the analyses for failing to complete the experiment (*n* = 1), responding in a language other than English (*n* = 1), or due to experimenter or equipment error (e.g., the testing session was not recorded [*n* = 2], or the participant's microphone quality interfered with the recording [*n* = 1]). We pilot tested 8 children to help us develop the task and coding scheme (their data is not included online or in any of the reported analyses).

Children were recruited from a database of volunteers from the Greater Metro‐Vancouver area in British Columbia. Parents and/or legal guardians were given the option to self‐report demographic information. Of parent/guardian‐reported data (78% of sample), 16 children were identified as East and Southeast Asian (27%), 15 as European (25%), 1 as South African (2%), 1 as South Asian (2%), and 1 as West Asian (2%). An additional 22% were identified as a combination of East and Southeast Asian and European (*n* = 7), European and Australian (*n* = 1), European and Canadian/USA (*n* = 1), European and South Asian (*n* = 3), and South Asian and Pacific Islander (*n* = 1).

The study was conducted during the COVID‐19 pandemic. Because of this, children were individually tested on a private Zoom video call with an experimenter. The experimenter's camera was turned on for the duration of the experiment, and caregivers hid their child's camera self‐view to minimize distraction. Experimenters were trained to not provide any cues or feedback to the child during the testing session. The experimenter reviewed the procedure with the child and their parent or guardian before sharing their screen, and caregivers confirmed that the stimuli were properly displayed before beginning the experiment. Consent was obtained from the parent or legal guardian present at the time of the study, and experimenters received verbal assent from children. Participants were compensated with a $5 gift card.

### 2.3 Stimuli and Design

As the experiment was conducted using Zoom, a video teleconference platform, participants completed the study from home on their computers. An experimenter ran a custom PsychoPy (Peirce et al. 2019) script via Pavlovia, a website for hosting and running online experiments, and shared their screen to display visual stimuli to accompany the verbal interview questions.

Each participant received the same 5 practice trials to familiarize them with the mode of response, followed by 24 test trials in randomized order. The complete list of questions is available in the Supplemental Materials (Tables S6 and S7). The practice trials are excluded from analyses. The entire procedure lasted between 15 and 40 min, depending on the speed at which the child responded.

**FIGURE 1:**
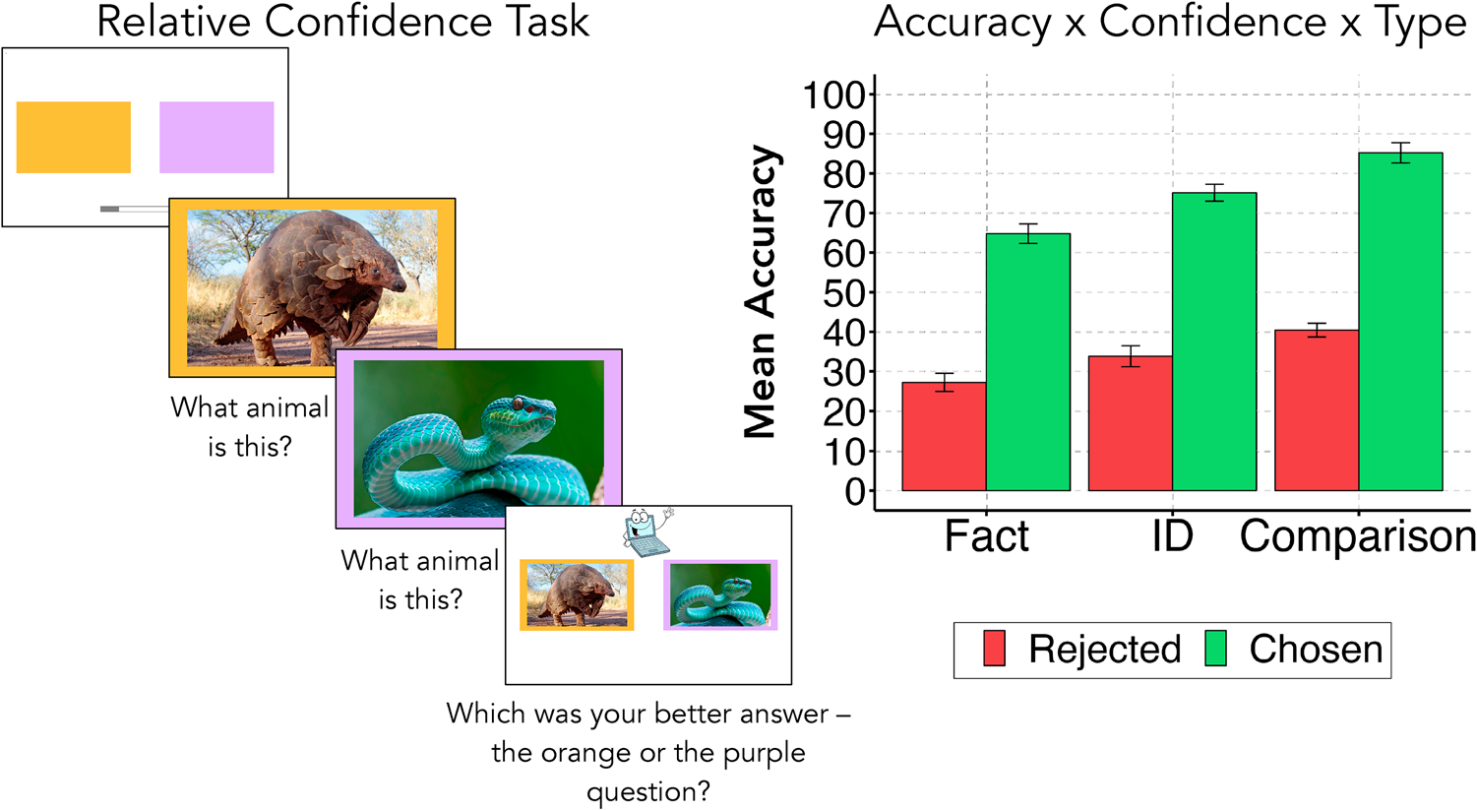
(Left) Example of the relative confidence task for an Animal ID question. The first trial is hard (“pangolin”) and the second trial is easy (“snake”). (Right) Accuracy rates across the three question types, by trial chosen (high confidence) versus trial rejected (low confidence).

Within a trial, participants were asked two questions, and then were asked to make a forced‐choice confidence judgment regarding which of their two answers was better (“Which was your best answer?”) (Figure 1; Baer and Odic 2019; Mamassian 2020). We included a range of question types (numerical comparison, animal identification/ID, animal fact) to examine explicit confidence judgments and verbal disfluency across domains. We bin these into two categories: Perceptual and Semantic. Numerical comparison questions (Perceptual) showed children images of nonsymbolic dot displays, with spatially separated collections of yellow dots on the left side of the screen and blue dots on the right, and asked them which of the two sides had more dots. Animal ID questions (Semantic) asked children to name an image of an animal, and animal fact questions (Semantic) required children to retrieve fact‐based knowledge from long‐term memory. We paired these questions so that—within a trial—the children answer two questions of the same type (e.g., two animal fact questions). We varied the difficulty of these paired questions in an effort to induce a range of confidence and certainty in their answers (e.g., within a trial, children are asked, “What are baby cats called?” and “What are baby swans called?” and are then asked which of their two answers they felt most confident about). The difficulty of these questions was estimated before the study and confirmed during pilot testing—we designed our questions so that children received the same number of predefined “easy,” “medium,” and “hard” questions, and these predefined bins were validated post hoc. While the order of the trials was randomized across participants, the question pairs within trials were consistent. Children's responses to the questions were recorded via Zoom and, as described below, fully transcribed and coded to estimate various disfluency cues.

### 2.4 Procedures

First, the experimenter explained the procedure to the child: they told the child that they would be answering a few different types of questions and emphasized that the child may not know the answer to all of them. The experimenter explained that the child should do their best to answer even when they are unsure. Each trial began with two colored response boxes on the screen: an orange box on the left side, and a purple box on the right (see Figure 1 for a trial schematic). Image stimuli were presented within the response boxes, and children used the colors to indicate their confidence decision. Then, the experimenter proceeded with the first practice trial. They displayed an image of a birthday cake, surrounded by the orange response box, and asked the child how old they are. Then, they displayed an image of a stranger, surrounded by the purple response box, and asked the child how old the stranger is. After the child answered both questions, the experimenter introduced the procedure for the forced‐choice confidence judgment. The images of the birthday cake and the stranger were displayed side by side. The birthday cake was again inside the orange response box, and the stranger inside the purple response box (thereby removing any memory load of matching question to the color), and children relied on these colors to verbally indicate their confidence choice. The experimenter explained that to “win” this game, the child needed to get a lot of questions right, and that to help the child out, they will allow the child to answer two questions and pick which of the two was their best answer, and they will choose this one for the computer to “check.” Then, they asked the child, “which of the two questions was their best answer, the one you're really sure you got right—the orange or the purple question?” The child indicated their response verbally by saying “purple” or “orange,” and the experimenter pressed a key to record their response. Then, the child answered 4 additional practice trials. All subsequent trials followed the same procedure, where the experimenter displayed an image associated with each question within the orange/purple response boxes, then after the child answered both questions in the pair, displayed the pair of images and asked the child to indicate which of the two was their better answer. These practice trials were designed to introduce the child to the various types of questions they will be answering during the test trials.

After the practice trials, participants completed the 24 test trials in random order, with intermixed question types, following the same procedure outlined above. Participants were permitted to skip any questions they did not know the answer to but were encouraged to provide their best guess. If the child was reluctant to answer, and did not provide a response within 15 s, the experimenter reminded them they could guess if they were unsure. If they did not answer after 30 s, the experimenter reminded them that they could say, “I don't know.” If the child ultimately didn't make a guess and responded with “I don't know,” we treated their answer as incorrect. If children skipped both knowledge questions (i.e., responded “I don't know” to both questions), the experimenter omitted the forced‐choice confidence judgment for that trial from any analysis involving confidence choice (2.43% of all trials).

### 2.5 Coding and Dependent Variables

**FIGURE 2:**
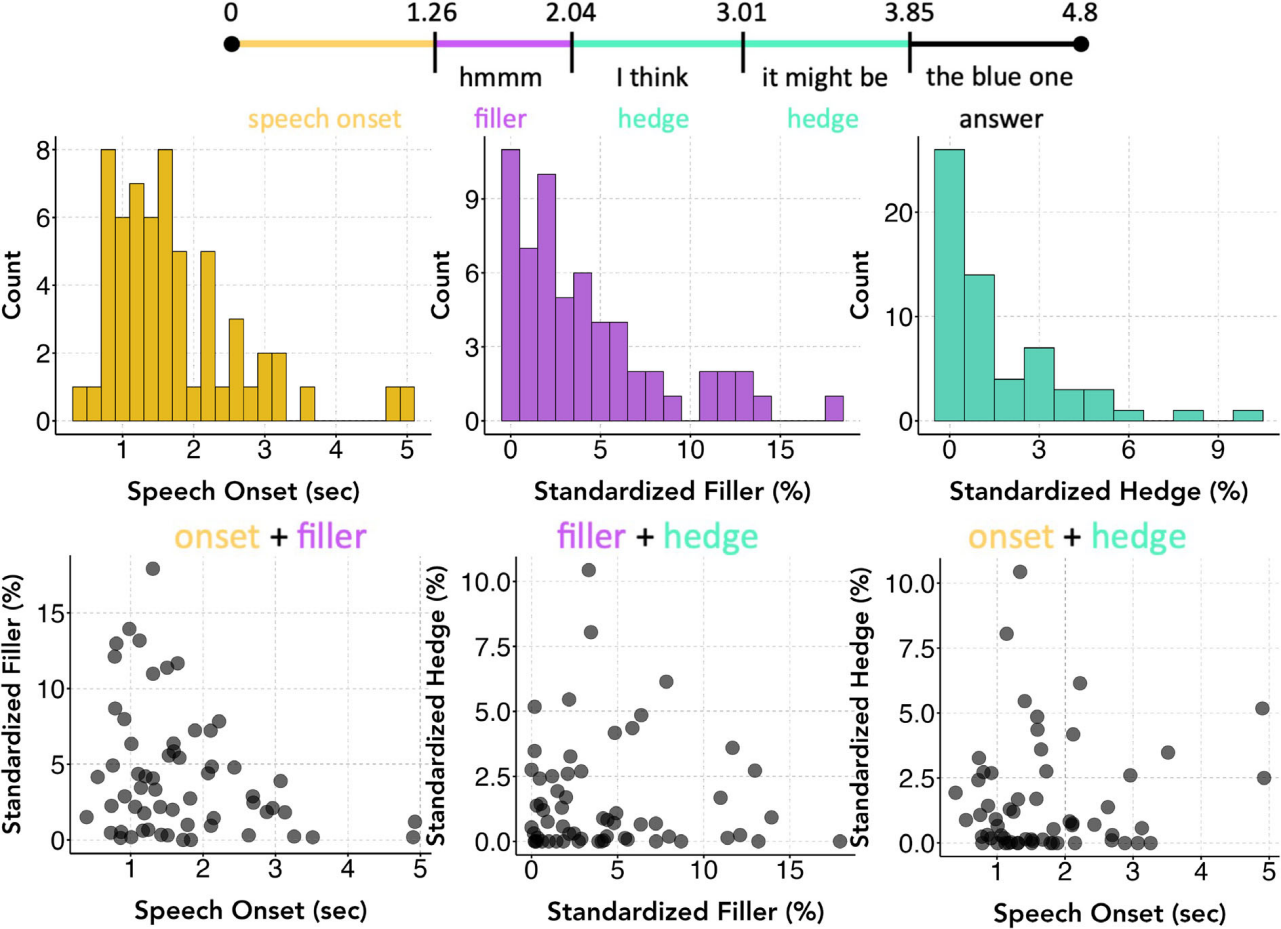
(Top) A transcript and coding of an example trial for numerical comparison (“which side has more dots?”). On this particular trial, the speech onset time would be 1.26 s, standardized filler would be 0.78/3.54 = 22%, and standardized hedge would be (0.97 + 0.84)/3.54 = 51%. Although this trial is from a real participant, it is unusual in the higher‐than‐average duration of both fillers and hedges and was chosen for illustrative purposes. (Bottom) The histograms for each of the three disfluency types, averaged at the participant level, as well as the pairwise correlations between them. Note that these pairwise plots do not regress out age, while our analyses do.

Verbal disfluency cues include speech onset (the delay before a participant begins answering a question), hedge phrases (e.g., “I think,” “maybe”), and fillers (e.g., “umm,” “mmm”; Bortfeld et al. 2001; Smith and Clark 1993). Consistent with the existing literature, we chose three primary measures of disfluency: *fillers*, *hedges*, and *speech onset time* (see Figure 2). Speech onset time is measured in seconds, while fillers and hedges are measured as a standardized duration—the total duration in seconds of fillers/hedges on a trial, divided by the total answer duration (i.e., trial duration minus speech onset time), expressed as a percent. Intuitively, a standardized filler duration of 50% means that half of the total period the child answered was spent making fillers. This standardization removes the confound of longer answers—especially the semantic ones—having more opportunities for fillers and hedges.

**FIGURE 3:**
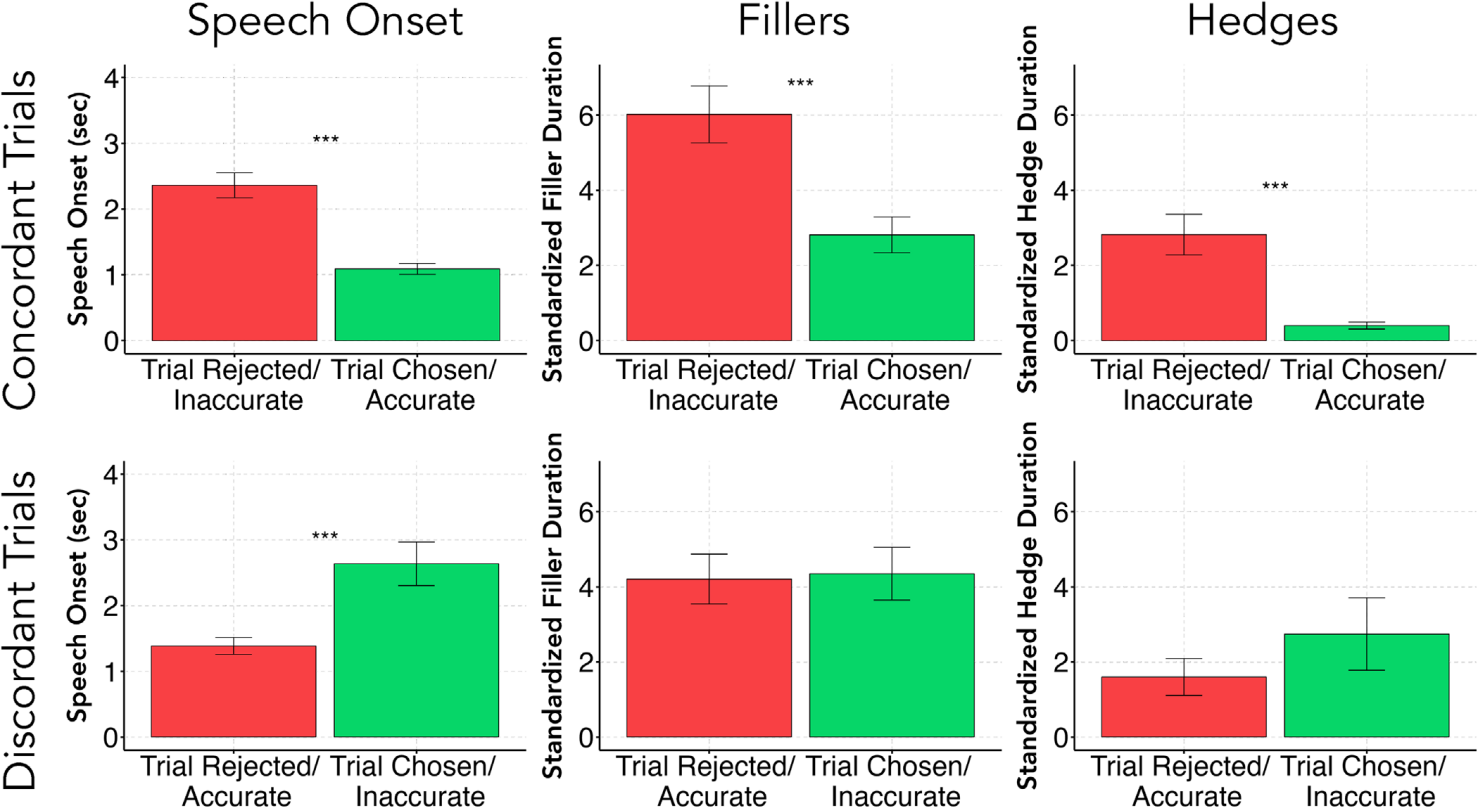
Bar graphs of verbal disfluency rates across chosen and rejected trials for concordant and discordant trials. While all three measures of disfluency predict confidence choice on concordant trials, with longer onsets, fillers, and hedges leading to rejection of trials, this pattern doesn't hold on discordant trials, where only speech onset predicts confidence choice, and in the opposite direction (longer speech onset is chosen).

Audio was recorded during the testing session using Zoom's audio recording feature. After testing, coders transcribed using the annotation software ELAN (Lausberg and Sloetjes 2009). Coders transcribed the child's response using a custom coding template, modeled after Smith and Clark's (1993) coding scheme and developed in detail using pilot data, which allowed them to record timestamps as well as categorize the child's answer into parts of speech of interest: fillers (e.g., “umm,” “uhh”), incomplete words (i.e., stammering), hedging phrases (e.g., “I think,” “maybe”), and reinforcing phrases (e.g., “I know,” “definitely”). Our analysis focuses only on fillers, hedges, and onset time, as these are more typically measured and manipulated in prior studies, but we make the coded data publicly available for researchers interested in patterns with other variables.

Coding and transcription were done in three steps. First, a trained coder transcribed and coded the data, and it was then reviewed by a second coder for any errors or alternative interpretations. Then, a third coder reviewed the trials with disagreements, and made the final decision on the coding (4% of trials from the first coder were changed by the third). Finally, 10% of the data was coded again by a fourth coder to calculate interrater reliability, which we found to be very high (ICC = 0.95, 95% CI [0.93, 0.95], *F*(431, 432) = 36.00, *p* < 0.001).

## 3 Results

### 3.1 Supplemental Materials

Due to the richness of the data, many analyses that are outside of the main questions of interest are presented in the Supplemental Materials. This includes analyses of disfluency production by gender, disfluencies produced during the confidence decision itself, the difference between “umm” versus “uhhh” to signal distinct metacognitive states (Clark and Fox Tree 2002; Fox Tree 2001), and more. Because we found few differences between semantic‐ and perceptually‐based decisions, we usually collapse them in the analyses reported here, but provide them as separate in the Supplemental Materials.

### 3.2 Analysis Plan

Our main analyses are carried out as multilevel logistic generalized mixed effects models, with either accuracy or confidence as binary outcome variables and the three measures of disfluency (Speech Onset, Standardized Filler Duration, Standardized Hedge Duration) and Age as fixed effects. Participants were included as random intercepts to account for individual variability while probing for general population‐level effects. Age was included in all models to control for expected developmental improvement in answer accuracy. To make sure that each measure of disfluency was meaningfully related to the outcome variable, we performed model comparison from a null model that only included the random effects and Age, and would successively add fixed effects, selecting the best‐fitting final model by a likelihood ratio test (i.e., “LRT‐selected models”). Estimates of each model's explained variance are presented with marginal (*R^2^_m_*) and conditional (*R^2^_c_*) *R^2^*, following Nakagawa and Schielzeth (2013). Marginal *R^2^_m_* quantifies the total proportion of variance explained by fixed effects alone, while conditional *R^2^_c_* represents the total variance explained by both fixed and random effects, allowing us to separate the contribution of fixed predictors from participant‐level variability. Model estimates are presented as odds ratios (ORs), with values above 1 indicating increases in the outcome variable with increases in the fixed effect, and values below 1 indicating increases in the outcome variable with decreases in the fixed effect. To control for family‐wise error, we performed a Holm‐Bonferroni correction. This resulted in two tests—flagged in the analyses with a * symbol—that meet the traditional alpha level of 0.05, but do not meet the corrected alpha level. Following the advice of an anonymous reviewer, post hoc power analyses were conducted for the primary GLMM analyses using the SIMR package in R (Green and MacLeod 2016). To estimate power to detect effects of disfluency for both accuracy and confidence, 1000 simulations were performed based on each LRT‐selected model. Post hoc power simulations confirmed 100% power to detect main effects of disfluency (Speech Onset, Standardized Filler Duration, Standardized Hedge Duration), and related interactions (Speech Onset × Concordance, Standardized Filler Duration × Concordance, Standardized Hedge Duration × Concordance) in predicting both confidence and accuracy.

### 3.3 Accuracy and Confidence

We first assess the accuracy of children's answers and the validity of the forced‐choice confidence judgment task, ignoring verbal disfluencies (Figure 1). To elicit a range of confidence, we designed questions that varied in difficulty. We evaluate whether these resulted in a range of responses and validate our predefined difficulty bins.

We examine the accuracy of children's answers by difficulty. Children, on average, answered 51.74% of questions correctly, 95% CI [49.06, 54.41]. Accuracy increased with age (*r*(58) = 0.51, *p <* 0.001). A sphericity‐corrected repeated‐measures ANOVA with Accuracy as the dependent variable and two factors (Difficulty: Easy/Medium/Hard and Question Type: Numerical Comparison/Animal ID/Animal Fact) revealed a significant main effect of Difficulty: participants provided more accurate answers for easier questions (Easy: *M* = 91.61%, 95% CI [89.56, 93.66]; Medium: *M* = 62.08%, 95% CI [57.73, 66.44]; Hard: *M* = 8.06%, 95% CI [5.50, 10.62]), *F*(2,118) = 1100.96, *p* < 0.001, *η*_p_^2^ = 0.80), and a main effect of Question Type (Numerical Comparison: *M* = 62.78%, 95% CI [60.71, 64.85]; Animal ID: *M* = 53.89%, 95% CI [50.65, 57.12]; Animal Fact: *M* = 45.08%, 95% CI [41.39, 48.78], *F*(2,118) = 53.68, *p* < 0.001, *η*_p_^2^ = 0.15). Finally, we observed an interaction between Difficulty and Question Type, *F*(4,236) = 70.49, *p <* 0.001, *η*_p_^2^ = 0.27. The main effect and interaction with question type is unsurprising, as numerical comparison questions are binary choices (blue or yellow), and guessing randomly would result in chance performance at 50% (except for the subset of impossible hard trials, which should pull overall chance to around 30%–40%).

Next, we examine if children reliably report their confidence in the forced‐choice task. If children reliably report their confidence in this task, their responses should track the accuracy of their answers; that is, their trial choice should predict their accuracy (Baer and Odic 2019; Mamassian 2020). A logistic generalized linear mixed effects model with participants as random effects, Accuracy as the outcome variable, and Trial Choice and Age as the fixed effects revealed a significant effect of Age (OR = 1.22, 95% CI [1.11, 1.34], z = 4.31, *p* < 0.001) and Trial Choice (OR = 5.62, 95% CI [4.76, 6.64], *z* = 20.38, *p* < 0.001). Fixed effects Trial Choice and Age explained 19.22% of the variance in Accuracy, and including participant‐level random effects accounted for 20.99% of variance (*R^2^_m_ = *.19; *R^2^_c_* = .21). Participants became more accurate with age, but also were more accurate on trials they chose, indicating higher confidence (M = 72.54%, 95% CI [68.65, 76.44]), than those they rejected, indicating lower confidence (M = 33.25%, 95% CI [30.05, 36.46]). As seen in Figure 1, this pattern held across all question types, as well. We observed no reliable primacy or recency effects across our sample (*t*(59) = −1.36, *p* = 0.18), though one child showed a tendency to choose the first/orange question on every trial they did not skip. Together, these results show that children's confidence was appropriately measured by the forced‐choice task, consistent with previous work (Baer and Odic 2019, 2020; Butterfield, Nelson, and Peck 1988).

We also examine age effects in confidence sensitivity by focusing on age‐related differences in concordance—the percent of trials in which children selected a correct answer as their confidence choice or rejected an incorrect answer as their choice, as opposed to discordant trials—those where they chose an incorrect answer or rejected a correct one^1^. Children were concordant on 69.64% of trials (95% CI [67.18, 72.11]), and this correlated with age (*r*(58) = 0.38, *p* = 0.003). Therefore, children were clearly able to select the better of the two answers, above chance (*t*(59) = 15.95, *p* < 0.001), and their ability to do this improved with age. As discussed in the Supplemental Materials, this age‐related effect was especially pronounced for the Perceptual questions and was absent for the Semantic questions.

### 3.4 Verbal Disfluency Production

Next, we assess children's overall production of disfluency, independent of its relation to accuracy and confidence. We also evaluate individual‐ and age‐related differences in verbal disfluency across the experiment.

We first examine the types and frequency of disfluency children produced. All participants produced at least one filler or hedge, and, of course, all children produced nonzero speech onset times. Fillers, treated as counts, were more common than hedges (Fillers: range = 0–45, *M =* 12.67, 95% CI [9.86, 15.48]; Hedges: range = 0–22, *M =* 4.93, 95% CI [3.40, 6.46]), although two children produced only hedges. Children most often used fillers “umm,” “uhh,” and “mmm” and hedges “I think” and “like.” Table S8 in the Supplemental Materials shows individual differences in the amount and type of disfluency, as well as descriptives for various other variables, such as average answer duration. We found no reliable age‐related effects after correcting for FWE (Speech Onset: *r*(58) = 0.26, *p =* 0.05*; Fillers: *r*(58) = −0.27, *p* = 0.04*; Hedges: *r*(58) = 0.20, *p* = 0.13). Therefore, from at least age 5 onward, children are reliable producers of disfluencies in all three categories.

We also evaluate whether different types of verbal disfluency are independent of each other. Figure 2 shows the histograms and pairwise plots for the three measures of disfluency. We found no correlations between the three measures of disfluency when controlling for age and FWE: Speech Onsets and Fillers (*r*(58) = −0.24; *p* = 0.07), Speech Onsets and Hedges (*r*(58) = 0.04; *p* = 0.77), and Fillers and Hedges (*r*(58) = 0.008; *p* = 0.95). Results were similar when using a Spearman correlation to account for the nonnormal distributions: Speech Onsets and Fillers (*r*(58) = −0.20, *p* = 0.13), Speech Onsets and Hedges (*r*(58) = −0.09, *p* = 0.50), and Fillers and Hedges (*r*(58) = 0.02, *p* = 0.87).

### 3.5 Disfluency and Accuracy

We next examine whether the three verbal disfluency types predict answer accuracy. Table 1 shows the average disfluency rates for Accurate and Inaccurate trials, showing that all three indices of disfluency predicted accuracy. Accuracy was significantly predicted by a model including any of the three indices of disfluency (compared to the null model), and the selected model, identified by hierarchical comparison with LRT, included all three categories as predictors (*χ* ^2^(1) = 31.91; *p* < 0.001). Given individual and age‐ differences in accuracy, we assess the proportion of variance in accuracy attributable to participants and calculate the intraclass correlation coefficient (ICC) from the null model. The observed ICC was .04, and fixed effects (Speech Onset, Standardized Filler Duration, Standardized Hedge Duration, Age) accounted for 18.62% of variance in Accuracy, while including random participant‐level effects explained 21.12% of variance (*R^2^_m_ =* 0.19; *R^2^_c_* = 0.21), suggesting individual differences have a limited effect on accuracy, and a larger portion of variance is explained by fixed predictors. VIF values were all below 1.02, suggesting minimal collinearity among the predictors. We observed a significant main effect of Age (OR = 1.27, 95% CI [1.15, 1.41], *z* = 4.87, *p* < 0.001), as older children provided more accurate answers. All categories of disfluency were negatively related with higher Accuracy: Speech Onset (OR = 0.72, 95% CI [0.68, 0.76], *z* = −11.68, *p* < 0.001), Standardized Filler Duration (OR = 0.98, 95% CI [0.97, 0.99], z = −5.50, *p* < 0.001), and Standardized Hedge Duration (OR = 0.97, 95% CI [0.95, 0.98], *z* = −4.82, *p* < 0.001). Therefore, children produce less verbal disfluency in all three categories when providing accurate answers, and more when providing inaccurate answers.

**TABLE 1:** Descriptive statistics for disfluency categories across accuracy and confidence judgments.

Accuracy	Accurate	Inaccurate
Speech onset	1.18 [1.01, 1.36]	2.37 [1.96, 2.77]
Standardized filler duration	3.19 [2.30, 4.08]	5.35 [4.00, 6.71]
Standardized hedge duration	0.74 [0.41, 1.08]	2.65 [1.67, 3.64]

Confidence	Higher confidence/Chosen	Lower Confidence/Rejected
Speech onset	1.43 [1.22, 1.64]	2.02 [1.71, 2.33]
Standardized filler duration	3.23 [2.21, 4.25]	5.40 [4.05, 6.74]
Standardized hedge duration	0.90 [0.55, 1.25]	2.33 [1.41, 3.25]

*Note*: Higher values for all disfluencies are associated with incorrect answers and rejected trials.

### 3.6 Disfluency and Confidence

Next, we evaluate whether children's disfluency predicts their confidence, independent of whether they answered correctly or not. Table 1 shows the average disfluency rates for Chosen (higher confidence) and Rejected (lower confidence) trials, with all three indices of disfluency clearly related to confidence. Confidence was significantly predicted by a model that includes any disfluency (compared to the null model), and the LRT‐selected model again included all three categories as predictors (*χ* ^2^(1) = 31.30; *p* < 0.001). Fixed effects (Speech Onset, Standardized Filler Duration, Standardized Hedge Duration, Age) accounted for 4.99% of variance in confidence (*R^2^_m_ =* 0.05). Including random intercepts for participants produced no measurable variance, resulting in a singular fit. Given the nature of the forced‐choice task, there was no participant‐level variability in confidence—50% of trials are necessarily chosen—so the conditional *R^2^_c_* could not be computed, and we observe no effects of Age in predicting Trial Choice (OR = 1.01, 95% CI [0.95, 1.08], *z* = 0.29 *p* = 0.77). VIF values were all below 1.03, suggesting minimal collinearity among the predictors. All categories of disfluency were negatively related to Trial Choice: Speech Onset (OR = 0.89, 95% CI [0.86, 0.92], *z* = −5.96, *p* < 0.001), Standardized Filler Duration (OR = 0.98, 95% CI [0.97, 0.99], *z* = −5.42, *p* < 0.001), and Standardized Hedge Duration (OR = 0.97, 95% CI [0.96, 0.98], *z* = −4.28, *p* < 0.001). Therefore, children produce more disfluency in all three categories while providing answers they feel less confident about, and less on answers they feel more confident about.

### 3.7 Disfluency and Concordance

Thus far, we have found that all categories of disfluency are predictive of children's confidence choice, making them a plausible cue for children to use in determining their confidence. However, as discussed in the Introduction, the correlations between disfluency and confidence could stem from various third variables, including answer accuracy or the difficulty of the questions. If disfluency itself is a cue to confidence, we should find that it predicts confidence choice even on discordant trials where confidence and accuracy are dissociated^2^.

First, we assess the interaction between disfluency and concordance in predicting confidence. We use the LRT‐selected confidence model reported above and additionally include concordance and its interaction with the three disfluency variables as fixed effects. Confidence was better predicted by this concordance interaction‐model (*χ* ^2^(4) = 220.74; *p* < 0.001). Fixed effects (Speech Onset, Standardized Filler Duration, Standardized Hedge Duration, Concordance, Age) and their interactions (Speech Onset × Concordance, Standardized Filler Duration × Concordance, Standardized Hedge Duration × Concordance) accounted for 20.35% of variance in confidence (*R^2^_m_ =* 0.20). Random effects again produced no measurable variance, due to the structure of the forced‐choice task, so conditional *R^2^_c_* was not computed, and we observe no effects of Age in predicting Trial Choice (OR = 1.02, 95% CI [0.95, 1.09], *z* = 0.49 *p* = 0.62). We find a significant main effect of Concordance (OR = 4.15, 95% CI [3.27, 5.28], *z* = 11.65, *p* < 0.001), and interaction with all three categories of disfluency: Speech Onset × Concordance (OR = 0.58, 95% CI [0.52, 0.64], *z* = −10.58, *p* < 0.001), Standardized Filler Duration × Concordance (OR = 0.96, 95% CI [0.94, 0.97], *z* = −5.19, *p* < 0.001), and Standardized Hedge Duration × Concordance (OR = 0.94, 95% CI [0.91, 0.96], *z* = −4.39, *p* < 0.001). This suggests the relationship between disfluency and confidence differs across concordant and discordant trials.

Table 2 and Figure 3 show the descriptive statistics for disfluency rates across concordant and discordant trials, split by confidence. For ease of interpretation, we report the main effects of disfluency from analyses that separate concordant and discordant trials. We find a strong relationship between disfluency and trial choice on concordant trials: increased verbal disfluencies again predicted lower confidence. The LRT‐selected model again included all three disfluency categories (*χ* ^2^(1) = 48.70; *p* < 0.001). Fixed effects (Speech Onset, Standardized Filler Duration, Standardized Hedge Duration, Age) explained 26.50% of the variance in Trial Choice, and including participant‐level random effects accounted for 26.73% of variance (*R^2^_m_ =* 0.27; *R^2^_c_* = 0.27). We observed a main effect of Age (OR = 1.20, 95% CI [1.10, 1.31], *z* = 4.21, *p* < 0.001), likely explained by the improvements in accuracy and concordance with age. Each category of disfluency was negatively related to Trial Choice: Speech Onset (OR = 0.69, 95% CI [0.64, 0.74], z = −9.95, *p* < 0.001), Standardized Filler Duration (OR = 0.97, 95% CI [0.96, 0.98], *z* = −6.52, *p* < 0.001), and Standardized Hedge Duration (OR = 0.94, 95% CI [0.91, 0.96], z = −5.45, *p* < 0.001). This replicates the patterns established above, which is unsurprising given that the majority of trials were concordant.

**TABLE 2:** Descriptive statistics for disfluency categories across concordant and discordant trials.

Concordant	Higher confidence/Chosen + Accurate	Lower confidence/Rejected + Inaccurate
Speech onset	1.09 [0.92, 1.25]	2.36 [1.98, 2.74]
Standardized filler duration	2.81 [1.86, 3.76]	6.02 [4.50, 7.54]
Standardized hedge duration	0.40 [0.22, 0.58]	2.82 [1.74, 3.90]

Discordant	Higher confidence/Chosen + Inaccurate	Lower confidence/Rejected + Accurate
Speech onset	2.64 [1.97, 3.31]	1.39 [1.13, 1.65]
Standardized filler duration	4.35 [2.93, 5.77]	4.21 [2.87, 5.54]
Standardized hedge duration	2.74 [0.79, 4.69]	1.60 [0.62, 2.58]

*Note*: While all three measures of disfluency predict confidence choice on concordant trials, with longer onsets, fillers, and hedges leading to rejection of trials, this pattern does not hold on discordant trials, where only speech onset predicts confidence choice, and in the opposite direction (longer speech onset is chosen).

On the other hand, we find that only the model with Speech Onset was a significant predictor of Trial Choice on discordant trials (*χ* ^2^(1) = 43.44; *p* < 0.001), and that adding Standardized Filler Duration and Standardized Hedge Duration did not improve the model any further (*χ* ^2^(2) = 0.31; *p* = 0.86). Fixed effects (Speech Onset, Age) explained 12.27% of the variance in Trial Choice, and including participant‐level random effects accounted for 15.76% of variance (*R^2^_m_ =* 0.12; *R^2^_c_* = 0.16). We observe a main effect of Age, though in the opposite direction (OR = 0.69, 95% CI [0.59, 0.80], *z* = −4.79, *p* < 0.001), again likely explained by the improvements in accuracy and concordance with age. Critically, the Speech Onset effect is *reversed* from what would be expected if children used it to determine confidence: children are more likely to choose the trial they *took longer* to begin answering (OR = 1.28, 95% CI [1.18, 1.40], *z* = 5.72, *p* < 0.001). Therefore, when separating accuracy and confidence, we find that verbal disfluencies fail to act as a predictor of confidence. As we elaborate in detail in the General Discussion, the pattern here is more consistent with short speech onsets predicting accuracy, not confidence.

Supplemental Materials show the above analysis with only the trial pairs that differed in accuracy, leading to the same conclusions for a link between fluency and confidence on concordant, but not discordant trials (Table S3). Supplemental Materials also present the above analyses split by Perceptual and Semantic questions (Table S2). In short, we find the concordant trials replicate the above results for both. Discordant Semantic trials also replicate the above analysis, with lower speech onset related to lower confidence. Discordant Perceptual trials, on the other hand, show no correlation with any measure of verbal fluency.

## 4 General Discussion

What is the relationship between fluency and confidence in young children? By relying on verbal disfluencies as a metric of fluency, we find that (1) English‐speaking children as young as five reliably produce an assortment of verbal disfluencies, including hedges and fillers, replicating previous work (Hübscher, Vincze, and Prieto 2019; Krahmer and Swerts 2005; Visser, Krahmer, and Swerts 2014), (2) their disfluencies predict accuracy and confidence equally well for both semantic and perceptual questions; but (3) higher fluency does *not* predict confidence on trials where confidence and accuracy dissociate (i.e., discordant trials). We find that hedges and fillers fail to predict confidence on discordant trials entirely, and while fast speech onset predicts high confidence on concordant trials, it predicts low confidence on discordant trials. As we elaborate below, our findings suggest that verbal disfluencies are therefore *not* used as a direct cue in children's confidence decisions.

Theories connecting fluency and confidence make a straightforward prediction: if fluency is a cue to confidence, then when fluency is high we should find high confidence, and when fluency is low we should find low confidence (e.g., Alter and Oppenheimer 2009; Koriat 1993). Concordant trials are consistent with this picture: when children responded faster, made fewer hedges and fillers, they indicated higher confidence than the reverse. But this pattern confounds fluency with other factors, including question difficulty and accuracy, which might affect both fluency and confidence independently. When examining discordant trials, we not only found that hedges and fillers do not predict confidence, but we also found a *reverse* effect for speech onset: fast responses were associated with low confidence, and slow ones with high confidence. Additionally, this pattern was primarily driven by the semantic questions, and we find no relationship at all between verbal disfluency cues and perceptual confidence on discordant trials (Supplemental Materials). This pattern challenges theories that argue that fluency and/or response times are causal cues for confidence decisions (e.g., Alter and Oppenheimer 2009; Koriat 1993).

How might we explain the pattern of results on discordant trials? One possibility is that children take verbal disfluency into account on all trials but somehow recognize that fast speech onset is sometimes a cue for high confidence and sometimes for low. For example, “fast errors” are a known pattern in the decision‐making literature, and often happen due to impulsive decision‐making, or after attentional lapses (Pleskac and Busemeyer 2010; Ratcliff and Rouder 1998). Perhaps children are aware that they sometimes make snap, impulsive decisions and—even if they answered correctly—subsequently indicate low confidence. Evidence from prior studies suggests children engage in response time monitoring, and associate fast errors with impulsivity: 5–8‐year‐olds who respond more rapidly before committing an error (a phenomenon known as pre‐error speeding) tend to show greater post‐error slowing, a behavior linked to cognitive control and error awareness (Ger and Roebers 2024). While nothing in our data precludes this possibility, theories now have to explain how children knew that they made an impulsive decision in the first place (and if children have access to that information, why would they subsequently take fluency into account at all?). Nevertheless, future theories on fluency might accommodate our results in such a manner.

The alternative explanation—and the one we prefer—is that speech onsets strongly relate to accuracy and are not necessarily incorporated into the subsequent confidence decision. Decisions that are “easy”—either because they are highly perceptually discriminable or reliably semantically accessible—will be made quickly and accurately, leading to short onsets. But children (and adults) might rely on an assortment of other cues to determine their confidence. Under the model of Pouget et al. (2016), for example, confidence is a mixture of representational signal strength (which correlates with fluency), trial history, one's belief in their abilities on tasks like these, evaluation of attentional states, and more. When all factors align—as they often do on concordant trials—verbal disfluencies will track confidence. However, when the factors misalign, the sum of various cues will result in a confidence decision that might vastly differ from the amount of fluency itself, as fluency is—at best—one of several cues attended to for this combination process.

Discordant trials, therefore, offer researchers an opportunity to separate the effects of different cues. To fully delineate a falsifiable cue‐based model of confidence, future work should strive to further isolate the effects of individual cues and determine which are necessary or sufficient for confidence decisions. One method to separate individual cues is to experimentally dissociate them. For example, some studies manipulate perceptual confidence, and reduce stimulus clarity with masking, while decision accuracy remains constant (Koizumi, Maniscalco, and Lau 2015). Dissociating these effects, experimentally, allows researchers more control and achieves a more balanced design where cues are necessarily in conflict. Isolating the independent contributions of different cues is also critical to determining the developmental trajectory of this process, to assess how individual cues are understood as children become better able to report their confidence. Some work suggests developmental improvement in the ability to integrate cues with their confidence decision, wherein older children's reported confidence better correlates with response time and accuracy (Koriat and Ackerman 2010). While our study was not designed to examine developmental change, and our current sample was not sufficiently powered to assess age‐related interactions, future work should explore how cue‐utilization develops and how different independent cues contributing to confidence interact with age. Investigating this relationship in younger children and toddlers—who are just beginning to produce language and verbal disfluency, and explicitly label and report their confidence—will be especially valuable for understanding its developmental origins. To identify the fundamental, necessary cues contributing to confidence, and to explain the emergence of reliable confidence judgments, a falsifiable cue‐based model must determine when and how children begin to integrate independent cues in their confidence decision.

None of this is to say that children don't use verbal disfluencies when judging the reliability of *others*, as a wealth of data has shown that they do (Birch, Akmal, and Frampton 2010; Jaswal and Malone 2007; Orena and White 2015; Richardson and Keil 2022). But this is because accessing information on reliability within ourselves is distinct from accessing that information in others (Baer, Malik, and Odic 2021). Given the abundance of internal cues we have about our thoughts and decision‐making process, we don't have to rely on fluency as the exclusive cue, or we can choose to disregard it entirely. When attending to others, however, we only have the observable data, and fluency is an especially prominent one, since we don't have access to other people's attentional states, representational strength, etc.

Beyond testing the fluency account of confidence, our work also significantly extends the existing literature on children's verbal disfluency production and its relationship to confidence more broadly. While some previous work has documented the presence of verbal disfluencies in young Catalan‐ and Dutch‐speaking children (Hübscher, Vincze, and Prieto 2019; Krahmer and Swerts 2005; Visser, Krahmer, and Swerts 2014), we extend this work to English‐speaking children. The coding templates used to transcribe the audio recordings of the test sessions, and the transcribed data files, are both available online. Transcribed files include not only each child's literal spoken words, but also categorize this speech into variables of interest, including hedge phrases, reinforcing phrases, fillers, incomplete words, repeated questions, and opt‐out or nonanswer phrases. We hope this will be useful for other researchers interested in further examining patterns of verbal disfluency.

We also are the first study of disfluency to include a relative confidence judgment. This approach has several advantages for our current study: first, it allowed us to easily separate concordant and discordant trials, which revealed differing patterns of results. Second, it removed the ability for children to respond overconfidently and indicate that they felt very sure about all of their answers. Recall that Hübscher, Vincze, and Prieto (2019) found that while preschoolers’ verbal disfluency predicted answer accuracy, it did not track their explicit confidence judgments as children often reported they felt “very sure.” In other words, there was little variability in their reported confidence. Here, children were required to choose which of two answers was *better*, and we were therefore able to separate high and low confidence—“chosen” compared to “rejected” questions. Although there are limitations to a relative confidence task—this binary judgment removes some granularity in Likert scale confidence judgments, for example—it is developmentally appropriate for children who may be “wishfully thinking”, and want to socially signal that they feel sure in their decisions (Schneider 1998), as well as for children who have little experience using such abstract scales.

Given mixed results in prior studies (e.g., Hübscher, Vincze, and Prieto 2019; Krahmer and Swerts 2005), it remains unclear whether these results would generalize to other contexts and designs, including single‐item Likert scale judgments. More work is needed to broaden the types of judgments and decisions in fluency studies, and separate task‐specific demand characteristics. It is important to acknowledge that judgments of certainty guide daily decisions in a broad range of contexts, and no single study can capture this full scope and complexity. Experimental design choices—such as question format (e.g., free recall vs. multiple choice), decision type (e.g., memory retrieval vs. perceptual), or domain (e.g., math vs. history), as well as the type of metacognitive judgment (e.g., relative 2AFC comparison vs. single‐item absolute judgments with Likert scales)—likely engage distinct cognitive and metacognitive processes, which may explain mixed results. To account for these variations, cue‐based models of confidence must explain which decision factors determine the cues used in a particular context.

Our findings also pose important implications for those trying to deduce children's accuracy, as in the case of eyewitness testimony. The pattern of speech onset predicting accuracy, not confidence, on discordant trials suggests that speech onset could be a more valuable, and more objective, metric for estimating children's accuracy. Interestingly, some previous findings that argue in favor of fluency as a cue to confidence using only correlational measures similarly report that reaction time was a more informative predictor of accuracy than confidence (Ackerman and Koriat 2011). This superiority of latency measures over confidence for estimating accuracy has similarly been suggested for adult eyewitnesses (Quigley‐McBride and Wells 2023), and our findings suggest that the same principle applies to children.

## Ethics Statement

This study was approved by the University of British Columbia's behavioral research ethics board (BREB H21‐00682). A parent or legal guardian was provided with a description of the study and gave written informed consent, and each participant provided verbal assent before participating.

## Conflicts of Interest

The authors declare no conflicts of interest.

## Supporting information



Supporting Information

## Data Availability

The data that support the findings of this study are openly available in Open Science Framework (OSF) at https://osf.io/6g2w4/?view_only=1d3179c016c2467395aef84f233d59aa, reference number 6g2w4.

## Endnotes

1 This analysis includes all trials—including pairs of trials where children answered both trials correctly or incorrectly. We chose to retain all trials to maximize power and because—even on two correct or incorrect trials—children may still be using their confidence choice to select the trial they were *more* confident in, making their selection a potentially meaningful variable even on these trials.

2 This analysis includes all trials—including pairs of trials where children answered both trials correctly or incorrectly. We chose to retain all trials to maximize power, because our conclusions did not change with them excluded. For example, if a child answered both questions correctly, a fluency account would still predict that the trial on which they produced *fewer* disfluencies should be the more confident one. In the Supplemental Materials, we also provide an additional set of analyses in which we only include trials on which children answered one question correctly, and the other incorrectly (Table S3). These analyses lead to identical conclusions to those presented here.

## References

[desc13617-bib-0001] Ackerman, R. , and A. Koriat . 2011. “Response Latency as a Predictor of the Accuracy of Children's Reports.” Journal of Experimental Psychology: Applied 17, no. 4: 406–417. 10.1037/a0025129. 21843018

[desc13617-bib-0002] Alter, A. L. , and D. M. Oppenheimer . 2009. “Uniting the Tribes of Fluency to Form a Metacognitive Nation.” Personality and Social Psychology Review 13, no. 3: 219–235. 10.1177/1088868309341564. 19638628

[desc13617-bib-0003] Ambrose, N. G. , and E. Yairi . 1999. “Normative Disfluency Data for Early Childhood Stuttering.” Journal of Speech, Language, and Hearing Research 42, no. 4: 895–909. 10.1044/jslhr.4204.895. 10450909

[desc13617-bib-0005] Baer, C. , I. K. Gill , and D. Odic . 2018. “A Domain‐General Sense of Confidence in Children.” Open Mind 2, no. 2: 86–96. 10.1162/opmi_a_00020.

[desc13617-bib-0050] Baer, C. , S. Ghetti , and D. Odic . 2021. Perceptual and Memory Metacognition in Children. Proceedings of the Annual Meeting of the Cognitive Science Society 43, no. 43. https://escholarship.org/uc/item/30g4w4sc.

[desc13617-bib-0006] Baer, C. , and C. Kidd . 2022. “Learning With Certainty in Childhood.” Trends in Cognitive Sciences 26, no. 10: 887–896. 10.1016/j.tics.2022.07.010. 36085134

[desc13617-bib-0007] Baer, C. , P. Malik , and D. Odic . 2021. “Are Children's Judgments of Another's Accuracy Linked to Their Metacognitive Confidence Judgments?” Metacognition and Learning 16, no. 2: 485–516. 10.1007/s11409-021-09263-x. 34720771 PMC8550463

[desc13617-bib-0008] Baer, C. , and D. Odic . 2019. “Certainty in Numerical Judgments Develops Independently of the Approximate Number System.” Cognitive Development 52: 100817. 10.1016/j.cogdev.2019.100817.

[desc13617-bib-0009] Baer, C. , and D. Odic . 2020. “Children Flexibly Compare Their Confidence Within and Across Perceptual Domains.” Developmental Psychology 56, no. 11: 2095. 10.1037/dev0001100. 32915050

[desc13617-bib-0010] Bayard, N. S. , M. H. van Loon , M. Steiner , and C. M. Roebers . 2021. “Developmental Improvements and Persisting Difficulties in Children's Metacognitive Monitoring and Control Skills: Cross‐Sectional and Longitudinal Perspectives.” Child Development 92, no. 3: 1118–1136. 10.1111/cdev.13486. 33529372 PMC8248442

[desc13617-bib-0011] Birch, S. A. J. , N. Akmal , and K. L. Frampton . 2010. “Two‐Year‐Olds Are Vigilant of Others' Non‐Verbal Cues to Credibility.” Developmental Science 13, no. 2: 363–369. 10.1111/j.1467-7687.2009.00906.x. 20136933

[desc13617-bib-0012] Bortfeld, H. , S. D. Leon , J. E. Bloom , M. F. Schober , and S. E. Brennan . 2001. “Disfluency Rates in Conversation: Effects of Age, Relationship, Topic, Role, and Gender.” Language and Speech 44, no. 2: 123–147. 10.1177/00238309010440020101. 11575901

[desc13617-bib-0013] Butterfield, E. C. , T. O. Nelson , and V. Peck . 1988. “Developmental Aspects of the Feeling of Knowing.” Developmental Psychology 24, no. 5: 654–663. 10.1037/0012-1649.24.5.654.

[desc13617-bib-0014] Clark, H. H. , and J. E. Fox Tree . 2002. “Using Uh and Um in Spontaneous Speaking.” Cognition 84, no. 1: 73–111. 10.1016/S0010-0277(02)00017-3. 12062148

[desc13617-bib-0015] DeJoy, D. A. , and H. H. Gregory . 1985. “The Relationship Between Age and Frequency of Disfluency in Preschool Children.” Journal of Fluency Disorders 10, no. 2: 107–122. 10.1016/0094-730X(85)90019-1.

[desc13617-bib-0016] Finn, B. , and J. Metcalfe . 2014. “Overconfidence in Children's Multi‐Trial Judgments of Learning.” Learning and Instruction 32: 1–9. 10.1016/j.learninstruc.2014.01.001.

[desc13617-bib-0017] Fox Tree, J. E. 2001. “Listeners' Uses of Um and Uh in Speech Comprehension.” Memory & Cognition 29, no. 2: 320–326. 10.3758/BF03194926. 11352215

[desc13617-bib-0018] Ger, E. , and C. M. Roebers . 2024. “Monitoring and Control Processes Within Executive Functions: Is Post‐Error Slowing Related to Pre‐Error Speeding in Children?” Journal of Experimental Child Psychology 246: 105975. 10.1016/j.jecp.2024.105975. 38852401

[desc13617-bib-0019] Geurten, M. , S. Willems , and M. Lloyd . 2021. “Too Much Familiarity! The Developmental Path of the Fluency Heuristic in Children.” Child Development 92, no. 3: 919–936. 10.1111/cdev.13449. 32808687

[desc13617-bib-0020] Green, P. , and C. J. MacLeod . 2016. “ simr: An R Package for Power Analysis of Generalized Linear Mixed Models by Simulation.” Methods in Ecology and Evolution 7, no. 4: 493–498. 10.1111/2041-210X.12504.

[desc13617-bib-0021] Grimaldi, P. , H. Lau , and M. A. Basso . 2015. “There Are Things That We Know That We Know, and There Are Things That We Do Not Know We Do Not Know: Confidence in Decision‐Making.” Neuroscience & Biobehavioral Reviews 55: 88–97. 10.1016/j.neubiorev.2015.04.006. 25929444 PMC4501881

[desc13617-bib-0022] Guttentag, R. , and J. Dunn . 2003. “Judgments of Remembering: The Revelation Effect in Children and Adults.” Journal of Experimental Child Psychology 86, no. 2: 153–167. 10.1016/S0022-0965(03)00135-8. 13129700

[desc13617-bib-0023] Halberda, J. , and L. Feigenson . 2008. “Developmental Change in the Acuity of the 'Number Sense': The Approximate Number System in 3‐, 4‐, 5‐, and 6‐Year‐Olds and Adults.” Developmental Psychology 44, no. 5: 1457–1465. 10.1037/a0012682. 18793076

[desc13617-bib-0025] Hübscher, I. , L. Vincze , and P. Prieto . 2019. “Children's Signaling of Their Uncertain Knowledge State: Prosody, Face, and Body Cues Come First.” Language Learning and Development 15, no. 4: 366–389. 10.1080/15475441.2019.1645669.

[desc13617-bib-0026] Jaswal, V. K. , and L. S. Malone . 2007. “Turning Believers Into Skeptics: 3‐Year‐Olds' Sensitivity to Cues to Speaker Credibility.” Journal of Cognition and Development 8, no. 3: 263–283. 10.1080/15248370701446392.

[desc13617-bib-0051] Kidd, C. , K. S. White , and R. N. Aslin . 2011. “Toddlers use Speech Disfluencies to Predict Speakers' Referential Intentions.” Developmental Science 14, no. 4: 925–934. 10.1111/j.1467-7687.2011.01049.x. 21676111 PMC3134150

[desc13617-bib-0027] Koizumi, A. , B. Maniscalco , and H. Lau . 2015. “Does Perceptual Confidence Facilitate Cognitive Control?” Attention, Perception, & Psychophysics 77, no. 4: 1295–1306. 10.3758/s13414-015-0843-3. 25737256

[desc13617-bib-0028] Koriat, A. 1993. “How Do We Know That We Know? The Accessibility Model of the Feeling of Knowing.” Psychological Review 100, no. 4: 609–639. 10.1037/0033-295X.100.4.609. 8255951

[desc13617-bib-0029] Koriat, A. , and R. Ackerman . 2010. “Choice Latency as a Cue for Children's Subjective Confidence in the Correctness of Their Answers.” Developmental Science 13, no. 3: 441–453. 10.1111/j.1467-7687.2009.00907.x. 20443965

[desc13617-bib-0030] Krahmer, E. , and M. Swerts . 2005. “How Children and Adults Produce and Perceive Uncertainty in Audiovisual Speech.” Language and Speech 48, no. 1: 29–53. 10.1177/00238309050480010201. 16161471

[desc13617-bib-0031] Kuhn, D. 2000. “Metacognitive Development.” Current Directions in Psychological Science 9, no. 5: 178–181. 10.1111/1467-8721.00088.

[desc13617-bib-0032] Lausberg, H. , and H. Sloetjes . 2009. “Coding Gestural Behavior With the NEUROGES‐ELAN System.” Behavior Research Methods 41, no. 3: 841–849. 10.3758/BRM.41.3.841. 19587200

[desc13617-bib-0033] Leckey, S. , D. Selmeczy , A. Kazemi , E. G. Johnson , E. Hembacher , and S. Ghetti . 2020. “Response Latencies and Eye Gaze Provide Insight on How Toddlers Gather Evidence Under Uncertainty.” Nature Human Behaviour 4, no. 9: 928–936. 10.1038/s41562-020-0913-y. 32690919

[desc13617-bib-0034] Ma, W. J. , and M. Jazayeri . 2014. “Neural Coding of Uncertainty and Probability.” Annual Review of Neuroscience 37, no. 1: 205–220. 10.1146/annurev-neuro-071013-014017. 25032495

[desc13617-bib-0035] Mamassian, P. 2020. “Confidence Forced‐Choice and Other Metaperceptual Tasks.” Perception 49, no. 6: 616–635. 10.1177/0301006620928010. 32552488

[desc13617-bib-0036] Nakagawa, S. , and H. Schielzeth . 2013. “A General and Simple Method for Obtaining R2 From Generalized Linear Mixed‐Effects Models.” Methods in Ecology and Evolution 4, no. 2: 133–142. https://pub.uni‐bielefeld.de/record/2565368.

[desc13617-bib-0037] Orena, A. J. , and K. S. White . 2015. “I Forget What That's Called! Children's Online Processing of Disfluencies Depends on Speaker Knowledge.” Child Development 86, no. 6: 1701–1709. 10.1111/cdev.12421. 26344559

[desc13617-bib-0038] Peirce, J. , J. R. Gray , S. Simpson , et al. 2019. “PsychoPy2: Experiments in Behavior Made Easy.” Behavior Research Methods 51, no. 1: 195–203. 10.3758/s13428-018-01193-y. 30734206 PMC6420413

[desc13617-bib-0047] Pleskac, T. J. , and J. R. Busemeyer . 2010. “Two‐Stage Dynamic Signal Detection: A Theory of Choice, Decision Time, and Confidence.” Psychological Review 117, no. 3: 864–901. 10.1037/a0019737. 20658856

[desc13617-bib-0048] Pouget, A. , J. Drugowitsch , and A. Kepecs . 2016. “Confidence and Certainty: Distinct Probabilistic Quantities for Different Goals.” Nature Neuroscience 19, no. 3: 366–374. 10.1038/nn.4240. 26906503 PMC5378479

[desc13617-bib-0039] Quigley‐McBride, A. , and G. L. Wells . 2023. “Eyewitness Confidence and Decision Time Reflect Identification Accuracy in Actual Police Lineups.” Law and Human Behavior 47, no. 2: 333–347. 10.1037/lhb0000518. 36757968

[desc13617-bib-0040] Rahnev, D. 2021. “Visual Metacognition: Measures, Models, and Neural Correlates.” American Psychologist 76, no. 9: 1445–1453. 10.1037/amp0000937. 35266744 PMC9295341

[desc13617-bib-0049] Ratcliff, R. , and J. N. Rouder . 1998. “Modeling Response Times for Two‐Choice Decisions.” Psychological Science 9, no. 5: 347–356. 10.1111/1467-9280.00067.

[desc13617-bib-0041] Richardson, E. , and F. C. Keil . 2022. “Thinking Takes Time: Children Use Agents' Response Times to Infer the Source, Quality, and Complexity of Their Knowledge.” Cognition 224: 105073. 10.1016/j.cognition.2022.105073. 35248759

[desc13617-bib-0042] Schneider, W. 1998. “Performance Prediction in Young Children: Effects of Skill, Metacognition and Wishful Thinking.” Developmental Science 1, no. 2: 291–297. 10.1111/1467-7687.00044.

[desc13617-bib-0043] Smith, V. L. , and H. H. Clark . 1993. “On the Course of Answering Questions.” Journal of Memory and Language 32, no. 1: 25–38. 10.1006/jmla.1993.1002.

[desc13617-bib-0044] Tian, Y. , T. Maruyama , and J. Ginzburg . 2017. “Self Addressed Questions and Filled Pauses: A Cross‐Linguistic Investigation.” Journal of Psycholinguistic Research 46, no. 4: 905–922. 10.1007/s10936-016-9468-5. 28028662

[desc13617-bib-0045] van Loon, M. , A. de Bruin , J. Leppink , and C. Roebers . 2017. “Why are Children Overconfident? Developmental Differences in the Implementation of Accessibility Cues When Judging Concept Learning.” Journal of Experimental Child Psychology 158: 77–94. 10.1016/j.jecp.2017.01.008. 28236719

[desc13617-bib-0046] Visser, M. , E. Krahmer , and M. Swerts . 2014. “Children's Expression of Uncertainty in Collaborative and Competitive Contexts.” Language and Speech 57, no. 1: 86–107. 10.1177/0023830913479117. 24754222

