# Supplementary material for: Do Young Children Use Verbal Disfluency as a Cue to Their Own Confidence?
Source: Dev Sci. 2025 Mar 5;28(3):e13617. doi: 10.1111/desc.13617 (PMC11883146; doi:10.1111/desc.13617)
Supplement: Supplementary file 1 — Supporting Information [file DESC-28-e13617-s001.docx]

**SUPPLEMENTAL RESULTS**

**Does confidence sensitivity change with age for both Semantic and Perceptual questions?**

We found that confidence sensitivity correlated with age – older children were more reliably able to select their better answer in the forced choice (*r*(58) = 0.38, *p* = 0.003). To further examine whether this age-related development in confidence sensitivity is symmetrical across semantic- and perceptually-based decisions, we separate question domains. Children selected the better answer on both Perceptual and Semantic questions: they were concordant on 72.36% of Perceptual questions (95% CI [68.47, 76.25]), and 68.80% of Semantic questions (95% CI [65.96, 71.63]). But while concordant choices correlated with age for Perceptual questions (*r*(58) = 0.53, *p* < 0.001)—older children more reliably selected the numerical comparison question they answered correctly—we observed no reliable correlation between concordance on Semantic questions and age (*r*(58) = 0.19, *p* = 0.14). To further probe the domain-specificity of confidence reasoning development, we examine whether individual differences in confidence sensitivity for Perceptual and Semantic questions are correlated. Individual differences in concordance across Perceptual and Semantic questions were uncorrelated (*r*(58) = 0.14, *p =* 0.30), suggesting children’s ability to select their better answers on Perceptual questions is independent from their ability on Semantic questions. This aligns with previous studies where individual differences in perceptual and memory confidence sensitivity dissociate, suggesting separable, domain-specific metacognitive processes underlie reasoning about perceptual decisions compared to decisions requiring retrieval from long term memory (Baer, Ghetti, et al., 2021).

**Are there differences in disfluency for Semantic and Perceptual questions?**

Children produced more verbal disfluency when answering incorrectly, and on low confidence trials. To examine whether this relationship between disfluency and accuracy, and disfluency and confidence differs between semantic- and perceptually-based decisions, we perform the same analyses separating question domains into Perceptual and Semantic.

*Relationship between Disfluency and Accuracy.* Supporting Materials Table S1 shows the average disfluency rates for Accurate and Inaccurate trials, separated by Perceptual and Semantic questions, showing that all three indices of disfluency predicted accuracy. Both Perceptual and Semantic decision accuracy was significantly predicted by a model including any of the three indices of disfluency (compared to the null model), and the LRT-selected model included all three categories as predictors (Perceptual questions: χ^2^(1) = 9.59; *p* = 0.002; Semantic questions: χ^2^(1) = 28.41; *p* < 0.001). VIF values were all below 1.03, suggesting minimal collinearity among predictors. For Perceptual questions, fixed effects (Speech Onset, Standardized Filler Duration, Standardized Hedge Duration, Age) accounted for 14.32% of variance in Accuracy (*R^2^_m_ =* 0*.*14). Including random intercepts for participants produced no measurable variance, resulting in a singular fit, so the conditional *R^2^_c_* could not be computed, and we observe no reliable effects of Age in predicting Accuracy (OR = 1.11, 95% CI [0.97, 1.27], *z* = 1.49 *p* = 0.14). This suggests little participant-level variability in accuracy on Perceptual questions. For Semantic Questions, fixed effects (Speech Onset, Standardized Filler Duration, Standardized Hedge Duration, Age) accounted for 18.94% of variance in Accuracy, and including random participant-level effects explained 22.26% of variance (*R^2^_m_ =* 0*.*19; *R^2^_c_* = 0.22). Accuracy on Semantic questions increased with Age (OR = 1.33, 95% CI [1.19, 1.49], *z* = 4.93, *p* < 0.001), suggesting age-related improvement in answer accuracy is largely driven by Semantic questions. All categories of disfluency were negatively related with higher Accuracy; Perceptual questions: Speech Onset (OR = 0.71, 95% CI [0.63, 0.80], *z* = -5.65, *p* < 0.001), Standardized Filler Duration (OR = 0.98, 95% CI [0.97, 0.99], *z* = -3.07, *p* = 0.002), Standardized Hedge Duration (OR = 0.96, 95% CI [0.92, 0.98], *z* = -2.85, *p* = 0.004); Semantic questions: Speech Onset (OR = 0.74, 95% CI [0.69, 0.78], *z* = -10.08, *p* < 0.001), Standardized Filler Duration (OR = 0.97, 95% CI [0.96, 0.98], *z* = -5.11, *p* < 0.001), Standardized Hedge Duration (OR = 0.97, 95% CI [0.95, 0.98], *z* = -3.82, *p* < 0.001). The relationship between verbal disfluency and accuracy, therefore, holds for both perceptual decisions and those requiring memory retrieval.

*Relationship between Disfluency and Confidence.* Supporting Materials Table S1 shows the average disfluency rates for Chosen and Rejected trials, with all three indices of disfluency clearly related to confidence for both domains. Confidence judgments, for both Perceptual and Semantic questions, were significantly predicted by a model that includes any disfluency (compared to the null model), and the LRT-selected model again included all three categories as predictors (Perceptual questions: χ^2^(1) = 10.11; *p* = 0.001; Semantic Questions: χ^2^(1) = 21.08; *p* < 0.001). Fixed effects (Speech Onset, Standardized Filler Duration, Standardized Hedge Duration, Age) accounted for 6.20% of variance in confidence (*R^2^_m_ =* 0*.*06) for Perceptual Questions, and 4.87% of variance (*R^2^_m_ =* 0*.*05) for Semantic Questions. Given the nature of the forced choice task, there was no participant-level variability in confidence – 50% of trials are necessarily chosen – so the conditional *R^2^_c_* could not be computed. We observe no effects of Age in predicting Trial Choice on Perceptual (OR = 0.98, 95% CI [0.86, 1.11], *z* = -0.33, *p* = 0.74) or Semantic questions (OR = 1.02, 95% CI [0.95, 1.10], *z* = 0.50, *p* = 0.62). VIF values were all below 1.03, suggesting minimal collinearity amongst the predictors. All categories of disfluency were negatively related with Trial Choice, Perceptual questions: Speech Onset (OR = 0.87, 95% CI [0.78, 0.96], *z* = -2.71, *p* = 0.007), Standardized Filler Duration (OR = 0.98, 95% CI [0.97, 0.99], *z* = -3.07, *p* = 0.002), Standardized Hedge Duration (OR = 0.96, 95% CI [0.92, 0.99], *z* = -2.56, *p* = 0.01); Semantic questions: Speech Onset (OR = 0.89, 95% CI [0.86, 0.93], *z* = -5.40, *p* < 0.001), Standardized Filler Duration (OR = 0.98, 95% CI [0.97, 0.99], *z* = -4.46, *p* < 0.001), Standardized Hedge Duration (OR = 0.98, 95% CI [0.96, 0.99], *z* = -3.48, *p* < 0.001). Thus, as with accuracy, the relationship between verbal disfluency and confidence holds for both perceptual decisions and those requiring memory retrieval.

Table S1: Descriptive statistics for disfluency categories across Accuracy and Confidence Judgments, and across Perceptual and Semantic question domains. Higher values for all disfluencies are associated with incorrect answers and rejected trials.

| **Accuracy** | **Perceptual Questions** | | **Semantic Questions** | |
| --- | --- | --- | --- | --- |
|  | Accurate | Inaccurate | Accurate | Inaccurate |
| **Speech Onset** | 1.09  [0.90, 1.29] | 1.85  [1.43, 2.26] | 1.22  [1.02, 1.42] | 2.50  [2.04, 2.96] |
| **Standardized Filler Duration** | 4.09  [2.63, 5.55] | 6.98  [4.54, 9.41] | 2.74  [1.98, 3.50] | 4.97  [3.75, 6.19] |
| **Standardized Hedge Duration** | 0.47  [0.04, 0.90] | 2.46  [0.85, 4.08] | 0.87  [0.47, 1.27] | 2.72  [1.60, 3.84] |
| **Confidence** | **Perceptual Questions** | | **Semantic Questions** | |
|  | Trial Chosen | Trial Rejected | Trial Chosen | Trial Rejected |
| **Speech Onset** | 1.19  [0.93, 1.44] | 1.57  [1.27, 1.87] | 1.51  [1.28, 1.74] | 2.17  [1.81, 2.53] |
| **Standardized Filler Duration** | 3.81  [2.23, 5.39] | 6.58  [4.50, 8.66] | 3.03  [2.11, 3.96] | 4.99  [3.74, 6.24] |
| **Standardized Hedge Duration** | 0.42  [0.02, 0.82] | 1.90  [0.62, 3.17] | 1.06  [0.64, 1.48] | 2.46  [1.37, 3.55] |

**Does the relationship between Concordance and Disfluency hold for both Semantic and Perceptual questions?**

To further examine whether the relationship between disfluency and confidence differs across domains, we separate Perceptual and Semantic questions and examine disfluency rates across concordant and discordant trials. First, we assess the interaction between disfluency and concordance in predicting confidence across Perceptual and Semantic question types. We use the LRT-selected confidence models reported above and additionally include concordance, and its interaction with the three disfluency variables, as fixed effects. For both Perceptual and Semantic question types, confidence was better predicted by this concordance interaction-model (Perceptual questions: χ^2^(4) = 106.18; *p* < 0.001; Semantic questions: χ^2^(4) = 160.45; *p* < 0.001). Fixed effects (Speech Onset, Standardized Filler Duration, Standardized Hedge Duration, Concordance, Age) and their interactions (Speech Onset x Concordance, Standardized Filler Duration x Concordance, Standardized Hedge Duration x Concordance) accounted for 24.35% of variance in Trial Choice on Perceptual questions (*R^2^_m_ =* 0*.*24), and 20.74% of variance in confidence on Semantic questions (*R^2^_m_ =* 0*.*21). Random effects again produced no measurable variance, due to the structure of the forced choice task, so Conditional *R^2^_c_* was not computed. We again observe no effects of Age in predicting Trial Choice on Perceptual (OR = 0.89, 95% CI [0.77, 1.02], *z* = -1.67, *p* = 0.10) or Semantic questions (OR = 1.04, 95% CI [0.96, 1.12], *z* = 1.01, *p* = 0.31). We find a significant main effect of Concordance across both question types (Perceptual questions: OR = 11.75, 95% CI [6.94, 20.69], *z* = 8.84, *p* < 0.001; Semantic questions: OR = 3.01, 95% CI [2.30, 3.96], *z* = 7.97, *p* < 0.001).

For Semantic Questions, we observe a significant interaction between concordance and disfluency on all three categories of disfluency: Speech Onset x Concordance (OR = 0.60, 95% CI [0.54, 0.67], *z* = -8.97, *p* < 0.001), Standardized Filler Duration x Concordance (OR = 0.94, 95% CI [0.92, 0.96], *z* = -5.33, *p* < 0.001), and Standardized Hedge Duration x Concordance (OR = 0.94, 95% CI [0.91, 0.97], *z* = -3.61, *p* < 0.001). In contrast, we observe a significant interaction between concordance and speech onset and hedges on Perceptual questions, but no interaction between concordance and fillers: Speech Onset x Concordance (OR = 0.56, 95% CI [0.43, 0.71], *z* = -4.34, *p* < 0.001), Standardized Filler Duration x Concordance (OR = 0.97, 95% CI [0.94, 1.01], *z* = -1.73, *p* = 0.08), and Standardized Hedge Duration x Concordance (OR = 0.91, 95% CI [0.84, 0.98], *z* = -2.39, *p* = 0.02). This suggests the relationship between disfluency and confidence differs across concordant and discordant trials for both Perceptual and Semantic question types.

Supporting Materials Table S2 shows the descriptive statistics for disfluency rates across concordant and discordant trials, split by confidence and by question domain. For ease of interpretation, we report main effects of disfluency from analyses which separate concordant and discordant trials. For concordant trials, across both Perceptual and Semantic domains, the LRT-selected model again included all three disfluency categories (Perceptual questions: χ^2^(1) = 14.81; *p* < 0.001; Semantic questions: χ^2^(1) = 39.43; *p* < 0.001). Fixed effects (Speech Onset, Standardized Filler Duration, Standardized Hedge Duration, Age) accounted for 20.61% of variance in confidence on Concordant Perceptual trials (*R^2^_m_ =* 0*.*21), and 27.74% of variance in confidence on Concordant Semantic questions (*R^2^_m_ =* 0*.*28). Including random participant-level effects produced no measurable variance, so Conditional *R^2^_c_* was not computed. We find no effect of Age on Concordant Perceptual questions (OR = 1.02, 95% CI [0.87, 1.20], *z* = 0.27 *p* = 0.79), but Trial Choice increased with Age for Concordant Semantic questions (OR = 1.26, 95% CI [1.14, 1.39], *z* = 4.68, *p* < 0.001), likely explained by the improvements in accuracy and concordance with age. Across Perceptual and Semantic domains, each disfluency category was negatively related to Trial Choice, Perceptual questions: Speech Onset (OR = 0.67, 95% CI [0.58, 0.77], *z* = -5.40, *p* < 0.001), Standardized Filler Duration (OR = 0.97, 95% CI [0.96, 0.99], *z* = -3.70, *p* < 0.001), Standardized Hedge Duration (OR = 0.92, 95% CI [0.86, 0.96], *z* = -2.88, *p* = 0.004); Semantic questions: Speech Onset (OR = 0.71, 95% CI [0.65, 0.76], *z* = -8.98, *p* < 0.001), Standardized Filler Duration (OR = 0.96, 95% CI [0.95, 0.97], *z* = -5.75, *p* < 0.001), Standardized Hedge Duration (OR = 0.94, 95% CI [0.91, 0.96], *z* = -4.61, *p* < 0.001). Therefore, concordance and disfluency are related for both domains and in an identical direction.

However, for discordant trials, we observe different results for Perceptual and Semantic domains. On discordant Perceptual trials, only Age predicted Trial Choice (OR = 0.50, 95% CI [0.35, 0.70], *z* = -3.85, *p* < 0.001). We observe no significant relationship between any measure of disfluency and Trial Choice: including Speech Onset, Standardized Filler Duration, and Standardized Hedge Duration, did not improve the null model that only included the random effects and Age (χ^2^(3) = 4.27; *p* = 0.23), and children produced similar levels of disfluency on chosen and inaccurate answers compared to rejected and accurate answers to numerical comparison questions.

For discordant Semantic questions, however, we find that the model with Speech Onset was a significant predictor of Trial Choice (χ^2^(1) = 31.12; *p* < 0.001), and that adding Standardized Hedge Duration and Standardized Filler Duration did not improve the model any further (χ^2^(2) = 1.41; *p* = 0.49). Fixed effects (Speech Onset, Age) accounted for 11.23% of variance in confidence on discordant Semantic trials (*R^2^_m_ =* 0*.*11) and including participant-level random effects explained 15.97% of variance (*R^2^_c_ =* 0*.*16). Age significantly predicted Trial Choice for discordant Semantic questions (OR = 0.68, 95% CI [0.57, 0.81], *z* = -4.32, *p* < 0.001). On discordant, Semantic questions, Speech Onset predicts trial choice in the opposite direction as would be expected if children use disfluency to determine confidence: children took longer to begin answering on chosen trials (OR = 1.26, 95% CI [1.15, 1.38], *z* = 4.88, *p* < 0.001). This suggests that the flipped effect of speech onset observed on discordant trials (Table 2) is primarily driven by Semantic questions.

Table S2: Descriptive statistics for disfluency categories across concordant and discordant trials and across Perceptual and Semantic question domains. While all three measures of disfluency predict confidence choice on concordant trials, for both Perceptual and Semantic questions, with longer onsets, fillers, and hedges leading to rejection of trials, this pattern doesn’t hold on discordant trials. On Perceptual discordant trials, no measure of disfluency predicts confidence, while for Semantic discordant trials, only speech onset predicts confidence choice, and in the opposite direction (longer speech onset is chosen).

| **Concordant** | **Perceptual Questions** | | **Semantic Questions** | |
| --- | --- | --- | --- | --- |
|  | Chosen + Accurate | Rejected + Inaccurate | Chosen + Accurate | Rejected + Inaccurate |
| **Speech Onset** | 1.05  [.86, 1.24] | 1.81  [1.41, 2.20] | 1.10  [0.92, 1.28] | 2.53  [2.08, 2.97] |
| **Standardized Filler Duration** | 3.65  [2.03, 5.27] | 8.37  [5.42, 11.31] | 2.46  [1.64, 3.28] | 5.44  [4.07, 6.81] |
| **Standardized Hedge Duration** | 0.22  [-0.06, 0.49] | 2.20  [0.68, 3.73] | 0.49  [0.25, 0.72] | 3.17  [1.76, 4.58] |
| **Discordant** |  | |  |  |
|  | Chosen + Inaccurate | Rejected + Accurate | Chosen + Inaccurate | Rejected + Accurate |
| **Speech Onset** | 1.75  [0.95, 2.54] | 1.19  [.93, 1.44] | 2.66  [1.98, 3.34] | 1.46  [1.13, 1.78] |
| **Standardized Filler Duration** | 4.25  [1.13, 7.36] | 4.65  [2.77, 6.54] | 4.52  [3.08, 5.97] | 3.94  [2.47, 5.40] |
| **Standardized Hedge Duration** | 2.47  [-1.24, 6.19] | 1.11  [0.00, 2.22] | 2.84  [0.86, 4.81] | 1.92  [0.62, 3.22] |

**Does the relationship between Concordance and Disfluency hold when excluding trials where both questions are answered correctly or incorrectly?**

Concordance analyses in the main text include pairs of trials where both questions in the pair are answered correctly or both incorrectly. In the forced choice confidence judgment task, when the accuracy of both answers in the pair are aligned, one trial is necessarily discordant – as the child must indicate which of their trials is *better*, they must either reject a correct answer (if both are answers in the pair were accurate) or choose an incorrect answer (if both answers in the pair were inaccurate). We include all trials in the main analyses to maximize power. Below, we report these same analyses, including *only* trial pairs where one answer was accurate, and one inaccurate.

We observe a similar pattern when excluding trial pairs where both answers were accurate or inaccurate. First, we assess the interaction between disfluency and concordance in predicting confidence. We replicate the LRT-selected confidence model reported in the main text, then the concordance-interaction model, and additionally include concordance, and its interaction with the three disfluency variables, as fixed effects. Confidence was better predicted by this concordance interaction-model (χ^2^(4) = 46.79; *p* < 0.001). Fixed effects (Speech Onset, Standardized Filler Duration, Standardized Hedge Duration, Concordance, Age) and their interactions (Speech Onset x Concordance, Standardized Filler Duration x Concordance, Standardized Hedge Duration x Concordance) accounted for 17.81% of variance in confidence (*R^2^_m_ =* 0*.*18). Random effects produced no measurable variance, due to the structure of the forced choice task, so Conditional *R^2^_c_* was not computed, and we observe no reliable effects of Age (OR = 1.06, 95% CI [0.97, 1.16], *z* = 1.30, *p* = 0.19). We find a significant main effect of Concordance (OR = 2.40, 95% CI [1.65, 3.50], *z* = 4.58, *p* < 0.001). We observe an interaction between concordance and speech onset and fillers on this subset of trials, but no significant interaction between concordance and hedges: Speech Onset x Concordance (OR = 0.62, 95% CI [0.52, 0.73], *z* = -5.66, *p* < 0.001), Standardized Filler Duration x Concordance (OR = 0.97, 95% CI [0.95, 1.00], *z* = -2.24, *p* = 0.03), and Standardized Hedge Duration x Concordance (OR = 0.97, 95% CI [0.93, 1.02], *z* = -1.41, *p* = 0.16).

Supporting Materials Table S3 shows the descriptive statistics for disfluency rates across concordant and discordant trials, split by confidence. For ease of interpretation, we report main effects of disfluency from analyses which separate concordant and discordant trials. We find a strong relationship between disfluency and trial choice on concordant trials: increased verbal disfluencies again predicted lower confidence. The LRT-selected model again included all three disfluency categories (χ^2^(1) = 28.12; *p* < 0.001). Fixed effects (Speech Onset, Standardized Filler Duration, Standardized Hedge Duration, Age) explained 20.25% of the variance in Trial Choice (*R^2^_m_ =* 0*.*20). Including random participant-level effects produced no measurable variance, so Conditional *R^2^_c_* was not computed, and we find no Age effects (OR = 1.08, 95% CI [0.98, 1.18], *z* = 1.51, *p* = 0.13). Each category of disfluency was negatively related to Trial Choice: Speech Onset (OR = 0.73, 95% CI [0.68, 0.79], *z* = -8.04, *p* < 0.001), Standardized Filler Duration (OR = 0.97, 95% CI [0.96, 0.98], *z* = -5.00, *p* < 0.001), and Standardized Hedge Duration (OR = 0.95, 95% CI [0.93, 0.97], *z* = -4.27, *p* < 0.001).

We also replicate the observed pattern on discordant trials when excluding pairs where one trial is necessarily discordant. We again find that only the model with Speech Onset was a significant predictor of Trial Choice on discordant trials (χ^2^(1) = 5.31; *p* = 0.02), and that adding Standardized Filler Duration and Standardized Hedge Duration did not improve the model any further (χ^2^(2) = 0.87; *p* = 0.65). Fixed effects (Speech Onset, Age) explained 2.61% of the variance in Trial Choice (*R^2^_m_ =* 0*.*03). Including random participant-level effects produced no measurable variance, so Conditional *R^2^_c_* was not computed, and we find no Age effects (OR = 0.98, 95% CI [0.79, 1.20], *z* = -0.24, *p* = 0.81). Critically, the Speech Onset effect is *reversed* from what would be expected if children used it to determine confidence: children are more likely to choose the trial they *took longer* to begin answering (OR = 1.17, 95% CI [1.02, 1.36], *z* = 2.19, *p* = 0.03). Therefore, when separating accuracy and confidence we find that verbal disfluencies fail to act as a predictor of confidence, and this is true both when including and excluding trial pairs where both are accurate or inaccurate.

Table S3: Descriptive statistics for disfluency categories across Concordant and Discordant trials, excluding trial pairs where both answers are accurate or inaccurate. While all three measures of disfluency predict confidence choice on concordant trials, with longer onsets, fillers, and hedges leading to rejection of trials, this pattern doesn’t hold on discordant trials, where only speech onset predicts confidence choice in the opposite direction (longer speech onset is chosen).

| **Concordant** |  | |
| --- | --- | --- |
|  | Chosen + Accurate | Rejected + Inaccurate |
| **Speech Onset** | 1.11  [0.93, 1.30] | 2.21  [1.82, 2.59] |
| **Standardized Filler**  **Duration** | 2.83  [1.73, 3.92] | 5.86  [4.22, 7.49] |
| **Standardized Hedge Duration** | 0.52  [0.28, 0.77] | 2.60  [1.55, 3.66] |
| **Discordant** |  | |
|  | Chosen + Inaccurate | Rejected + Accurate |
| **Speech Onset** | 1.82  [1.35, 2.28] | 1.39  [1.00, 1.79] |
| **Standardized Filler Duration** | 3.67  [1.81, 5.52] | 4.94  [2.27, 7.60] |
| **Standardized Hedge**  **Duration** | 1.72  [-0.33, 3.77] | 1.52  [-0.45, 3.50] |

**Did children produce disfluencies when making the confidence decision itself?**

Children provided their confidence judgments by verbally reporting which of the two paired questions was their better answer. We additionally transcribed the speech produced during confidence judgments and assess disfluency rates produced during the confidence choice. Children produced less disfluency during the confidence choice compared to their answers to test questions: fourteen participants produced no fillers, and 42 participants produced no hedge phrases while reporting their confidence. Fillers, treated as counts, were more common than hedges (Fillers: range = 0-17, *M =* 4.22*,* 95% CI [3.11, 5.33]; Hedges: range = 0-10, *M =* 0.95*,* 95% CI [0.43, 1.47]). We found weak age-related effects (Speech Onset: *r*(58) = -0.01, *p =* 0.94; Fillers: *r*(58) = -0.41, *p* = 0.001; Hedges: *r*(58) = 0.18, *p* = 0.18), with older children tending to produce fewer fillers, but maintaining similar onsets and producing a similar number of hedges as younger children when judging confidence.

To further examine whether reasoning about confidence differs across domains, we compare disfluency produced during the confidence choice across Perceptual and Semantic questions. Children produced more disfluency when judging their confidence on Semantic questions (Fillers: range = 0-13, *M =* 3.08*,* 95% CI [2.23, 3.94]; Hedges: range = 0-7, *M =* 0.78*,* 95% CI [0.37, 1.20]) compared to Perceptual questions (Fillers: range = 0-5, *M =* 1.13*,* 95% CI [0.78, 1.49]; Hedges: range= 0-3, *M =* 0.17*,* 95% CI [0.03, 0.30]); Fillers: (*t*(78.88) = 4.20, *p* < 0.001); Hedges: (*t*(71.36) = 2.81, *p* = 0.006); although they produced similar onsets across domains during the confidence judgment (*t*(116.33) = 0.25, *p* = 0.80).

**Do children produce disfluencies differently when choosing to not answer a question?**

Although children were encouraged to guess on every trial, some children chose to skip trials that they did not know the answer to. We assess whether disfluency rates differ across answered questions and those where children provided at least one non-answer phrase (e.g., “I don’t know” or “skip”). The model that included Speech Onset and Standardized Hedge Duration significantly predicted non-answers (χ^2^(1) = 14.97; *p* < 0.001), but the addition of Standardized Filler Duration did not improve the model any further (χ^2^(1) = 0.13; *p* = 0.72). Fixed effects (Speech Onset, Standardized Hedge Duration, Age) explained 8.73% of the variance in Trial Choice, and including participant-level random effects accounted for 33.87% of variance (*R^2^_m_ =* 0*.*09; *R^2^_c_* = 0.34). We observe no reliable Age effects (OR = 0.94, 95% CI [0.70, 1.27], *z* = -0.45, *p* = 0.66), suggesting children provided similar rates of non-answers across development. While children took longer to begin answering when they ultimately provided a non-answer (OR = 1.11, 95% CI [1.04, 1.17], *z* = 3.41, *p* < 0.001), the effect of hedges was the opposite direction: children produced *fewer* hedges on unanswered trials (OR = 0.92, 95% CI [0.83, 0.97], *z* = -2.39, *p* = 0.02; Supporting Materials Table S4). The amount of filler disfluencies did not differ across answered and unanswered trials.

Table S4: Disfluency rates by answer type (answered vs. unanswered trials)

|  | **Answered Questions** | **Unanswered Questions** |
| --- | --- | --- |
| **Speech Onset** | 1.69  [1.44, 1.93] | 2.15  [1.60, 2.70] |
| **Standardized Filler** | 4.27  [3.14, 5.40] | 3.39  [1.99, 4.79] |
| **Standardized Hedge** | 1.69  [1.09, 2.29] | 0.53  [0.06, 1.00] |

**Are there differences in disfluency rates by gender?**

We examine gender differences in the amount of disfluency produced while answering questions (i.e., ignoring disfluencies during confidence decisions). Gender was collected by a parent-administered free response questionnaire (N = 32 boys, N = 27 girls, N = 1 non-binary participant; excluded from gender analyses). We observed no effect of gender: girls and boys produced similar rates of fillers (*t*(53.35) = -1.07, *p* = 0.28) and hedges, treated as counts, (*t*(49.60) = -1.36, *p* = 0.18), and comparable speech onsets (*t*(55.80) = 0.59, *p* = 0.56; Supporting Materials Table S5).

Table S5: Disfluency rates by gender. Filler and hedge counts are summed across all test trials.

|  | **Boys** | **Girls** |
| --- | --- | --- |
| **N Fillers** | 10.81  [7.33, 14.30] | 13.67  [9.48, 17.85] |
| **N Hedges** | 3.84  [1.96, 5.73] | 5.96  [3.38, 8.54] |
| **Mean Speech Onset** | 1.80  [1.45, 2.15] | 1.65  [1.28, 2.02] |

**Duration of pauses following “umm” vs. “uhh”**

Smith & Clark (1993) found a distinction between “umm” and “uhh”: adults used “uhh” to signal short delays, and “umm” for longer ones. Thus, we examine the duration of the pause following “umms” and “uhhs”. A linear mixed-effects model, including Filler Type (umm, uhh) and Age as fixed effects, and participants as random intercepts, revealed no effect of Filler Type. Including Filler Type did not improve model fit relative to the null model which included only random effects of Age (χ^2^(1) = 2.59; *p* = 0.11). We find no reliable effects of Age (OR = 1.35, 95% CI [0.89, 2.06], *t*(18.71) = 1.39, *p* = 0.18), suggesting no developmental change in the duration of pauses following disfluency. Children did not reliably produce different durations of pauses following “umms” (M = 1.81, 95% CI [0.19, 3.43]) and “uhhs” (M = 2.30, 95% CI [0.23, 4.36]) (OR = 0.53, 95% CI [0.25, 1.15], *t*(156.12) = -1.63, *p* = 0.11), as found in 3- and 4-year-old children (Hudson Kam & Edwards, 2008)

**SUPPLEMENTAL TABLES**

Table S6: Practice questions by Question Type and Difficulty Bin. “Answer” column indicates the accepted answer for each question. Accuracy

rates are across children. Accuracy rates are binned by ratio for the numerical comparison questions.

| **Question Type** | **Question** | **Answer** | **Difficulty Bin** | **Accuracy Rate** |
| --- | --- | --- | --- | --- |
| Age | \| How old are you? \| \| --- \| | NA | Easy | 100.00% |
| Age | How old is this person? | NA | Hard | 0.00% |
| Numerical Comparison | Which side has more dots? (6 vs. 24) | 24 | Easy | 95.83% |
| Numerical Comparison | Which side has more dots? (12 vs. 18) | 18 | Mid | 90.00% |
| Numerical Comparison | Which side has more dots? (15 vs. 15) | Neither | Hard | 1.67% |
| Animal ID | What kind of animal is this? | Groundhog | Hard | 8.33% |
| Animal ID | What kind of animal is this? | Squirrel | Easy | 90.00% |
| Animal Fact | What sound does a lion make? | Roar | Easy | 91.67% |
| Animal Fact | What sound does a hyena make? | Hehehe | Mid | 24.14% |

Table S7: Test questions by Question Type and Difficulty Bin. “Answer” column indicates the accepted answer for each question. Accuracy rates are across children. Accuracy rates are binned by ratio for the numerical comparison questions.

| **Question Type** | **Question** | **Answer** | **Difficulty bin** | **Accuracy** |
| --- | --- | --- | --- | --- |
| Animal Fact | What are baby dogs called? | Puppies | Easy | 77.05% |
| Animal Fact | What are baby cats called? | Kittens | Easy | 80.00% |
| Animal Fact | What animal has the longest neck in the world? | Giraffe | Easy | 80.00% |
| Animal Fact | What do cows eat? | Grass | Easy | 83.33% |
| Animal Fact | What color is a pig? | Pink | Easy | 86.67% |
| Animal Fact | What color is a dolphin? | Grey | Easy | 60.00% |
| Animal Fact | What sound does a horse make? | Neigh | Easy | 75.00% |
| Animal Fact | What sound does a cat make? | Meow | Easy | 90.00% |
| Animal Fact | What are baby swans called? | Cygnets | Hard | 0.00% |
| Animal Fact | What are baby llamas called? | Cria | Hard | 0.00% |
| Animal Fact | What animal has the longest tongue in the world? | Anteater | Hard | 13.33% |
| Animal Fact | What color is an octopus' blood? | Blue | Hard | 26.67% |
| Animal Fact | What color is a hippo's milk? | Pink | Hard | 16.67% |
| Animal Fact | What do blue whales eat? | Krill | Hard | 15.00% |
| Animal Fact | What sound does a zebra make? | Heehaw | Hard | 6.67% |
| Animal Fact | What sound does a cheetah make? | Chirping | Hard | 8.47% |
| Animal Fact | \| What are baby deer called? \| \| --- \| | Fawns | Mid | 6.67% |
| Animal Fact | What are baby sheep called? | Lambs | Mid | 40.00% |
| Animal Fact | What animal has the biggest ears in the world? | Elephant | Mid | 60.00% |
| Animal Fact | What color is a polar bear's skin underneath it's fur? | Black | Mid | 38.33% |
| Animal Fact | What color is a robin's egg? | Blue | Mid | 36.67% |
| Animal Fact | What do koalas eat? | Eucalyptus leaves | Mid | 63.33% |
| Animal Fact | What sound do fish make? | Glub glub | Mid | 70.00% |
| Animal Fact | What sound does a dolphin make? | Whistle/  clicking | Mid | 48.33% |
| Animal ID | What kind of animal is this? | Snake | Easy | 98.33% |
| Animal ID | What kind of animal is this? | Frog | Easy | 100.00% |
| Animal ID | What kind of animal is this? | Rabbit | Easy | 100.00% |
| Animal ID | What kind of animal is this? | Dog | Easy | 98.33 % |
| Animal ID | What kind of animal is this? | Pangolin | Hard | 10.00% |
| Animal ID | What kind of animal is this? | Capybara | Hard | 15.00% |
| Animal ID | What kind of animal is this? | Axolotl | Hard | 15.00% |
| Animal ID | What kind of animal is this? | Elephant shrew | Hard | 6.67% |
| Animal ID | What kind of animal is this? | Ferret | Mid | 11.67% |
| Animal ID | What kind of animal is this? | Hamster | Mid | 63.33% |
| Animal ID | What kind of animal is this? | Platypus | Mid | 56.67% |
| Animal ID | What kind of animal is this? | Hedgehog | Mid | 71.67% |
| Numerical Comparison | Which side has more dots? (6 vs. 24) | 24 | Easy | 96.67% |
| Numerical Comparison | Which side has more dots? (15 vs. 15) | Neither | Hard | 1.67% |
| Numerical Comparison | Which side has more dots? (12 vs. 18) | 18 | Mid | 90.00% |

Table S8: Individual differences in the number of fillers, hedges, and non-answer phrases produced while answering questions, and the average duration of provided answers. Filler, hedge, and non-answer counts do not include disfluency produced while reporting confidence. Mean answer durations are the calculated from speech offset—the total time from the end of the experimenter’s question to the end of the child’s speech—and include latencies to begin answering. Total experiment duration is the total duration of the recorded testing session.

| **Participant Number** | **N Fillers** | **N Hedges** | **N Non-Answers** | **Mean Answer Duration**  **(sec)** | **Total Experiment Duration**  **(min:sec)** |
| --- | --- | --- | --- | --- | --- |
| **1** | 17 | 2 | 9 | 3.82 | 21:35 |
| **2** | 33 | 6 | 0 | 4.38 | 19:23 |
| **3** | 2 | 2 | 1 | 3.82 | 19:06 |
| **4** | 8 | 20 | 2 | 3.65 | 18:50 |
| **5** | 8 | 0 | 0 | 3.54 | 21:44 |
| **6** | 15 | 1 | 4 | 3.86 | 19:51 |
| **7** | 11 | 13 | 0 | 3.67 | 21:43 |
| **8** | 28 | 4 | 0 | 6.33 | 20:26 |
| **9** | 4 | 1 | 3 | 3.80 | 17:48 |
| **10** | 45 | 12 | 5 | 5.60 | 23:23 |
| **11** | 2 | 1 | 0 | 3.11 | 19:00 |
| **12** | 1 | 0 | 0 | 2.70 | 19:38 |
| **13** | 11 | 0 | 3 | 3.43 | 20:34 |
| **14** | 13 | 17 | 12 | 8.46 | 20:35 |
| **15** | 29 | 1 | 1 | 4.19 | 21:31 |
| **16** | 10 | 1 | 7 | 3.43 | 19:23 |
| **17** | 23 | 15 | 8 | 6.00 | 20:15 |
| **18** | 3 | 5 | 1 | 2.87 | 15:59 |
| **19** | 19 | 20 | 4 | 4.69 | 17:32 |
| **20** | 18 | 22 | 0 | 6.62 | 29::27 |
| **21** | 12 | 7 | 18 | 3.90 | 29:14 |
| **22** | 12 | 0 | 5 | 5.17 | 21:31 |
| **23** | 25 | 4 | 2 | 3.51 | 15:51 |
| **24** | 0 | 5 | 5 | 3.14 | 14:20 |
| **25** | 1 | 0 | 15 | 2.77 | 16:55 |
| **26** | 13 | 15 | 1 | 3.94 | 18:45 |
| **27** | 10 | 2 | 5 | 5.48 | 21:51 |
| **28** | 6 | 13 | 0 | 3.37 | 17:35 |
| **29** | 5 | 3 | 0 | 2.97 | 17:45 |
| **30** | 2 | 0 | 10 | 5.70 | 20:15 |
| **31** | 14 | 1 | 0 | 4.01 | 17:44 |
| **32** | 10 | 3 | 6 | 3.90 | 21:31 |
| **33** | 28 | 8 | 3 | 7.44 | 26:29 |
| **34** | 19 | 0 | 0 | 3.43 | 19:12 |
| **35** | 36 | 0 | 0 | 3.88 | 21:41 |
| **36** | 3 | 5 | 0 | 2.55 | 12:34 |
| **37** | 39 | 2 | 0 | 5.65 | 16:43 |
| **38** | 0 | 1 | 13 | 3.88 | 21:59 |
| **39** | 1 | 4 | 0 | 3.49 | 16:17 |
| **40** | 17 | 4 | 3 | 4.46 | 18:37 |
| **41** | 4 | 0 | 3 | 2.79 | 17:59 |
| **42** | 12 | 9 | 2 | 5.17 | 24:31 |
| **43** | 16 | 2 | 2 | 4.27 | 11:48 |
| **44** | 21 | 0 | 3 | 5.85 | 20:01 |
| **45** | 3 | 0 | 1 | 3.08 | 15:02 |
| **46** | 5 | 3 | 7 | 4.15 | 16:27 |
| **47** | 9 | 1 | 0 | 2.74 | 17:00 |
| **48** | 21 | 0 | 10 | 2.81 | 20:13 |
| **49** | 9 | 1 | 3 | 2.61 | 23:37 |
| **50** | 6 | 0 | 0 | 4.25 | 21:02 |
| **51** | 1 | 11 | 1 | 5.83 | 20:29 |
| **52** | 28 | 12 | 2 | 5.14 | 21:55 |
| **53** | 31 | 2 | 7 | 2.57 | 17:46 |
| **54** | 2 | 2 | 0 | 1.97 | 15:59 |
| **55** | 1 | 4 | 19 | 5.03 | 28:15 |
| **56** | 6 | 2 | 2 | 4.68 | 23:21 |
| **57** | 8 | 3 | 4 | 3.83 | 19:41 |
| **58** | 16 | 14 | 2 | 3.54 | 22:10 |
| **59** | 5 | 10 | 2 | 6.73 | 22:02 |
| **60** | 3 | 0 | 4 | 2.92 | 19:31 |
